# Establishment of highly metastatic KRAS mutant lung cancer cell sublines in long-term three-dimensional low attachment cultures

**DOI:** 10.1371/journal.pone.0181342

**Published:** 2017-08-07

**Authors:** Tomoyuki Nakano, Yoshihiko Kanai, Yusuke Amano, Taichiro Yoshimoto, Daisuke Matsubara, Tomoki Shibano, Tomoko Tamura, Sachiko Oguni, Shizuka Katashiba, Takeshi Ito, Yoshinori Murakami, Masashi Fukayama, Takashi Murakami, Shunsuke Endo, Toshiro Niki

**Affiliations:** 1 Division of General Thoracic Surgery, Jichi Medical University, Shimotsuke, Tochigi, Japan; 2 Division of Integrative Pathology, Jichi Medical University, Shimotsuke, Tochigi, Japan; 3 Molecular Pathology Laboratory, Institute of Medical Science, University of Tokyo, Tokyo, Japan; 4 Department of Human Pathology, Graduate School of Medicine, University of Tokyo, Tokyo, Japan; 5 Department of Microbiology, Faculty of Medicine, Saitama Medical University, Moroyama, Saitama, Japan; Technische Universitat Dresden, GERMANY

## Abstract

Decreased cell-substratum adhesion is crucially involved in metastasis. Previous studies demonstrated that lung cancer with floating cell clusters in histology is more likely to develop metastasis. In the present study, we investigated whether cancer cells in long-term, three-dimensional low attachment cultures acquire high metastatic potential; these cells were then used to examine the mechanisms underlying metastasis. Two KRAS-mutated adenocarcinoma cell lines (A549 and H441) were cultured and selected on ultra-low attachment culture dishes, and the resulting cells were defined as FL (for floating) sublines. Cancer cells were inoculated into NOD/SCID mice via an intracardiac injection, and metastasis was evaluated using luciferase-based imaging and histopathology. *In vitro* cell growth (in attachment or suspension cultures), migration, and invasion were assayed. A whole genomic analysis was performed to identify key molecular alterations in FL sublines. Upon detachment on low-binding dishes, parental cells initially formed rounded spheroids with limited growth activity. However, over time in cultures, cells gradually formed smaller spheroids that grew slowly, and, after 3–4 months, we obtained FL sublines that regained prominent growth potential in suspension cultures. On ordinary dishes, FL cells reattached and exhibited a more spindle-shaped morphology than parental cells. No marked differences were observed in cell growth with attachment, migration, or invasion between FL sublines and parental cell lines; however, FL cells exhibited markedly increased growth potential under suspended conditions *in vitro* and stronger metastatic abilities *in vivo*. A genomic analysis identified epithelial-mesenchymal transition (EMT) and c-Myc amplification in A549-FL and H441-FL cells, respectively, as candidate mechanisms for metastasis. The growth potential of FL cells was markedly inhibited by lentiviral ZEB1 knockdown in A549-FL cells and by the inhibition of c-Myc through lentiviral knockdown or the pharmacological inhibitor JQ1 in H441-FL cells. Long-term three-dimensional low attachment cultures may become a useful method for investigating the mechanisms underlying metastasis mediated by decreased cell-substratum adhesion.

## Introduction

Lung cancer is the leading cause of cancer mortality in the United States, Europe, Japan, and other developed and developing countries [1]. The prognosis of lung cancer patients is generally poor; successfully treated patients with stage IA lung cancer have a 5-year survival rate of only 80% after complete surgical resection [2]. Metastasis, the transfer of cancer from one part of the body to another not directly connected part, is the major cause of cancer-related deaths [3]. According to a previous study [4], the risk of loco-regional and distant relapse in stage I lung cancer remains high at 22–30%. Approximately 55% of patients newly diagnosed with non-small cell lung cancer (NSCLC) have distant metastasis [5]. Therefore, metastatic tumor models are essential for developing new therapeutic methods to cure lung cancer patients.

Among the four major histological subtypes of lung cancer, the increased incidence of adenocarcinoma is widely recognized [6]. Adenocarcinomas have five major architectural growth patterns; lepidic, papillary, acinar, solid, and micropapillary patterns [7]. The presence of a small percentage of micropapillary and solid growth patterns is a strong predictor of a poor prognosis and an increased risk of recurrence [7–10]. The micropapillary pattern is characterized by papillary structures with tufts lacking a central fibrovascular core that float in alveolar spaces or stroma [9].

Cancer cell clusters floating in tissue, as exemplified in MPC, have been suggested to accommodate an environment in which cancer cells are deprived of substrate adherence. Although decreased cell adhesion is generally considered to play a role in tumor metastasis [11], the mechanisms by which floating cancer cells acquire high metastatic potential need to be validated in an experimental model.

KRAS is one of the commonly mutated oncogenes in lung cancer, while activated KRAS mutations are detected in 15–30% of patients with NSCLC and predict a poor prognosis in response to treatment regimens [12,13]. Effective therapies including standard chemotherapy to target lung cancer with mutated KRAS have not yet been developed [14].

In the present study, we exposed two KRAS-mutant lung adenocarcinoma cell lines, A549 and NCI-H441, to long-term, 3-dimensional (3D) cultures on low-binding culture dishes. This approach allowed us to obtain sublines, designated as FL (for floating), that acquired the ability to steadily grow under suspended conditions. Metastatic potential was higher in these FL cells than in parental cell lines. Furthermore, through a molecular analysis, we identified possible molecular mechanisms that confer growth potential to FL cells under detached conditions.

## Materials and methods

### Cell lines and culture conditions

The human KRAS-mutated lung cancer cell lines, A549 and NCI-H441 (hereafter H441) were obtained from the American Type Culture Collection (ATCC, Rockville, MD, USA). Cell lines were maintained and grown in RPMI 1640 (Sigma-Aldrich, St. Louis, NJ, USA) supplemented with 10% fetal bovine serum (FBS; Autogen-Bioclear, Wiltshire, UK), 100 U/mL penicillin, and 100 μg/mL streptomycin in a humidified atmosphere of 5% CO_2_ and 95% air.

### Antibodies and reagents

Rabbit polyclonal anti-Poly ADP-ribose polymerase (PARP) (#9542), anti-cleaved PARP (#9541), and anti-cyclin B1 (#4138P) antibodies and mouse monoclonal anti-cyclin A2 (#4656P; clone BF683) and anti-p21 (#2946P; clone DCS60) antibodies were obtained from Cell Signaling Technology (Danvers, MA, USA). Rabbit polyclonal anti-cyclin D1 (RM9104-S1) antibodies was obtained from Thermo Fisher Scientific (Waltham, MA, USA). A rabbit polyclonal anti-zinc finger E-box binding homeobox 1 (ZEB1) (HPA028524) antibody and mouse polyclonal anti-beta actin (A5441) antibody were obtained from Sigma-Aldrich. A rabbit monoclonal anti-p27 antibody (ab32034, clone Y236) was obtained from Abcam (Cambridge, MA, USA). Mouse monoclonal anti-cyclin E1 (SC-247, clone HE12), anti-p16 (SC-1661; clone F-12), and anti-c-Myc (SC-40; clone 9E10) antibodies were obtained from Santa Cruz Biotechnology (Santa Cruz, CA, USA). A mouse monoclonal anti-E-cadherin (610181; clone 36/E-Cadherin) antibody was obtained from BD Bioscience (Franklin Lakes, NJ, USA). A mouse monoclonal anti-vimentin (M0725, clone V9) antibody was obtained from DAKO (Glostrup, Denmark). JQ1 (1268524-70-4) was obtained from Cayman Chemical (Ann Abor, MI, USA). Lentiviral sh-RNA vectors were from Dharmacon (Lafayette, CO, USA).

### Long-term low attachment culture

In order to selectively establish cell lines with debased cell-substratum adhesion, cell lines (A549 and H441) were placed in a 100-mm Ultra-Low Attachment Culture Dish (Corning, Corning, NY, USA) coated with a covalently bonded hydrogel surface that was hydrophilic and uncharged under the same culture conditions described above. Two cell lines were cultured in ultra-low attachment dishes for three or four months until the cell cluster became loose and cell growth was stable in the low attachment culture. These cells were defined as the FL subline (A549-FL, H441-FL).

### Cell growth assay

Cell growth was analyzed using Cell Counting Kit-8 (CCK-8) (Dojindo Molecular Technology, Kumamoto, Japan). Briefly, 5 × 10^2^ cells (A549, A549-FL) and 2 × 10^3^ cells (H441, H441-FL) in RPMI with 10% FBS were seeded on a 96-well normal attachment microtiter plate or a 96-well Ultra-Low Attachment Surface microtiter plate (Corning) with quintuplicate repeats for each condition. After the indicated time for the incubation, CCK-8 reagent was added to each well and incubated for 4 hours. The formazan dye formed by viable cells was measured at 450 nm for absorbance values with a reference at 630 nm using a BIO-RAD model 680XR microplate reader (Bio-Rad, Hercules, CA, USA).

### DNA quantitative analysis

A total of 1 × 10^5^ cells (A549, A549-FL) and 2 × 10^5^ cells (H441, H441-FL) in RPMI with 10% FBS were seeded on 60-mm Ultra-Low Attachment Culture Dishes (Corning). Immediately after plating (day 0) or after a 7-day incubation, cells were collected by centrifugation, lysed by lysis buffer, and DNA was extracted by the standard protocol using proteinase K digestion and phenol/chloroform extraction. The concentrations of DNA solutions were calculated by measuring absorbance at 260 nm using Nano EX (Optima, Schaumburg, IL, USA).

### Migration assay

Transwell cambers with 8-μm pores were obtained from Corning. Cells were harvested and re-suspended in RPMI with 10% FBS at concentrations of 2 × 10^5^ cells (A549, A549-FL) and 4 × 10^5^ cells (H441, H441-FL) in 1 mL, and then seeded into the upper chamber of a 12-well plate. The lower chambers were filled with 2.3 mL RPMI containing 10% FBS. Cells were incubated for 2 days. At the end of the experiment, cells that migrated into the reverse side of the transwell membrane were fixed with methanol, stained with Giemsa stain solution, and then counted under an inverted light microscope.

### Matrigel invasion assay

In the tumor cell invasion assay, transwell chambers with 8-μm pores and transwell membranes pre-coated with Matrigel (BD Bioscience) were obtained from Corning. Cells were harvested and re-suspended in RPMI with 10% FBS at concentrations of 1 × 10^5^ cells (A549, A549-FL) and 2 × 10^5^ cells (H441, H441-FL) in RPMI with 10% FBS in 0.5 mL, and then seeded into the upper chamber of a 24-well plate. The lower chambers were filled with 1.6 mL RPMI containing 10% FBS. Cells were incubated for 2 days. At the end of the experiment, cells that had invaded into the reverse side of the transwell membrane were fixed with methanol, stained with Giemsa stain solution, and then counted under an inverted light microscope.

### Western blot analysis

Cells were lysed by sonication in radioimmunoprecipitation assay (RIPA) buffer consisting of 20 mmol/L Tris-HCL (pH7.4),150 mmol/L NaCl, 50 mmol/L NAF, and 1 mmol/L Na_3_VO_4_ with a cocktail of proteinase inhibitors. Lysates were then immersed in water at 98°C for five minutes and cleared by centrifugation. Protein concentrations were measured using the DC Protein Assay kit (Bio-Rad). In the Western blot analysis, equal amounts of protein samples were separated by size on 8% polyacrylamide gels and electroblotted onto a nitrocellulose membrane. Non-specific binding was blocked by the immersion of membranes at room temperature for 30 minutes in 5% skim milk in Tris-buffer saline (TBS). Membranes were washed with TBS buffer containing 0.1% Tween 20, incubated at room temperature for one hour with primary antibodies, washed, and then reacted with peroxidase-conjugated secondary antibodies. We detected the antigen using ECL Western Blotting Detection Reagents (Amersham, GE Healthcare Life Science, Little Chalfont, Buckinghamshire, UK) in order to confirm protein expression, and protein levels were assessed by Western blotting in accordance with the manufacturer’s instructions. We used ChemiDoc XRS system of BioRad to capture gel images and quantify intensities of the bands.

### Retroviral transduction of the firefly luciferase vector

Firefly (*Photinus pyralis*) luciferase cDNA from pGL3 basic (Promega, Madison, WI, USA) was inserted into the pMSCV puro retro-viral vector (Clontech, Palo Alto, CA, USA), generating pMSCV-luciferase. GP2-293 packaging cells (Clontech) were co-transfected with pMSCV-luciferase and pVSV-G (Clontech), a plasmid encoding the viral envelope glycoprotein (VSV-G) of vesicular stomatitis virus, using Lipofectamine 2000 (Invitrogen, Carlsbad, CA, USA) according to the manufacturer’s instructions. Supernatants from transfected GP2-293 were induced with approximately 50% confluent cells in the presence of polybrene (8 μg/ml final concentration; Sigma-Aldrich). The transduced cells were propagated in medium containing puromycin (Sigma-Aldrich) at 15 μg/ml and the generated cells were named luc-A549, luc-A549-FL, luc-H441, and luc-H441-FL. Cell morphology and growth characteristics of the cells were unchanged after transduction of the luciferase gene (S1 Fig). Before the *in vivo* experiment, the luciferase activity of each cell type was measured *in vitro* and found to be similar.

### Intracardiac tumor injection and *in vivo* bioluminescence imaging

Female NOD.CB-17-Prkdc^scid^/J (NOD/SCID) mice were obtained from Charles River Japan (Yokohama, Japan). Animal experiments in this study were approved by the Animal Ethics Review Board of Jichi Medical University (No. 16151) and were performed in accordance with the Institutional Guides for Laboratory Animals and the Principles of Laboratory Animals Car formulated by the National Society for Medical Research. All mice were housed in plastic cages containing wood shavings for bedding and allowed free access to water and a chew diet in a 12-hour light/dark cycle, with room temperature at 23 ± 2°C. For experimental manipulation, mice were anesthetized by inhalation of 2% isoflurane, which was delivered by an anesthetic vaporizer. Cells were harvested by trypsinization and washed twice in PBS before being injected. Cells (2.5 × 10^5^ /200 μL) were injected using a 30-gauge needle into the left cardiac ventricle of anesthetized NOD/SCID mice (8 weeks old, n = 5 per group) under ultrasound (US) guidance with the Vevo770 high-resolution US system (Visual Sonics, Toronto, Ontario, Canada).

*In vivo* tumor metastasis was examined using the non-invasive bioimaging system IVIS^™^ (Xenogen, Alameda, CA, USA). D-luciferin (1 mg/body; Biosynth, Staad, Switzerland) was injected into the peritoneal cavity of anesthetized tumor-implanted mice. The resulting gray scale photographic and pseudocolor luminescent images were automatically superimposed using software to facilitate the identification of any optical signal and location on the mouse. Signals from tumors were quantified as a photon flux (photons/s/cm^2^/steradian). Examinations were performed on a weekly basis from two weeks after the injection. Model mice were examined for clinical signs, distress, decreased physical activity, and body weight twice a week.

### Sacrifice and organ harvest

Model mice were sacrificed after eight weeks or were immediately sacrificed when weight loss of 20% or more occurred and/or the maximum tumor size was larger than 10 mm in order to prevent suffering. Mice were deep anesthetized by inhalation of 4% isoflurane and euthanized by manual cervical dislocation. Death of animals was confirmed by cessation of respiration. Two mice died prior to sacrifice. Because the mice had metastatic disease at the time of death, we presume that cause of death was metastasis. In all experiments the maximum tumor size was less than 5 mm. Thirteen organs (brain, teeth, lungs, stomach, liver, pancreas, spleen, kidneys, adrenal gland, ovaries, bowels, spine, and femurs) were dissected, cut into 2.0-mm-thick sections for paraffin embedding, and stained with hematoxylin and eosin (H&E) in order to evaluate metastasis under a light microscope. The extent of metastasis was histopathologically evaluated using H&E-stained sections of the dissected organs with a semi-quantitative scoring system based on the size and number of lesions (Table 1).

**Table 1:** Score and criteria in the semi-quantitative evaluation of murine metastasis models.

score	criteria
**0**	No metastasis
**1**	< 0.2 mm and < 3 lesions
**2**	< 0.2 mm and ≥ 3 lesions
	0.2–2.0 mm and < 3 lesions
**3**	0.2–2.0 mm and ≥ 3 lesions
	> 2.0 mm and < 3 lesions
**4**	> 2.0 mm and ≥ 3 lesions

### Gene expression profiling and genomic copy number analysis

Total DNA and RNA were isolated from four cell lines (A549, A549-FL, H441, and H441-FL) using the Qiagen DNeasy Blood & Tissue Kit and RNeasy Plus Mini Kit, respectively, according to the manufacturer’s instructions (Qiagen, Valencia, CA, USA). DNA was analyzed using the Agilent Sure Print G3 Human comparative genomic hybridization (CGH) 4 × 180K kit, and RNA was analyzed using the Agilent Sure Print G3 Human gene expression (GE) 8 × 60K Ver. 2.0 kit at the Biomedical Center of Takara Bio Inc., Mie, Japan.

A Gene Set Enrichment Analysis (GSEA) was used to investigate the enriched functions of specifically expressed genes [15]. The specifically expressed gene list was ranked according to fold changes and compared with sets of genes that were classified according to all oncogenic signature gene sets. Results with a P-value ≤0.05 and false discovery rate (FDR) ≤0.25 were considered to be significant.

### Lentiviral knockdown (KD) of ZEB1 and c-Myc

In order to assess the roles of ZEB1 and c-Myc, we knocked down their expression using TRC lentiviral shRNA vectors. We selected two plasmids of ZEB1 shRNA; ZEB1-A (TRCN0000017564; mature antisense sequence 5’-AATCGTTTGCTTATTGGCAGC-3’) and ZEB1-B (TRCN0000017567; mature antisense sequence 5’-AACTGTTGGCAGAACAACAGC-3’), and two plasmids of c-Myc shRNA; c-Myc-A (TRCN0000039642; mature antisense sequence 5’-TTGTTGCTGATCTGTCTCAGG-3’) and c-Myc-B (TRCN0000174055; mature antisense sequence 5’-TTGTTGCTGATCTGTCTCAGG-3’). The frameworks of these shRNAs were formed by the pLKO.1 cloning vector (#10878, Addgene, Cambridge, MA, USA). 293T packaging cells (RIKEN, Tsukuba, Japan) were seeded on 60-mm dishes, and were co-transfected with shRNA plasmids, the pCMV-VSV-G and pCMV-dR8.2 dvpr vectors (Addgene, Cambridge, MA, USA) using Lipofectamine 2000 (Invitrogen, Carlsbad, CA, USA) to produce functional lentiviral particles. The vector containing a random DNA sequence was used as a negative control (shScramble). A549 and A549-FL cells were transduced with shZEB1-A, shZEB1-B, and shScramble; A549, A549-FL, H441, and H441-FL cells were transduced with shc-Myc-A, shc-Myc-B, and shScramble. After a brief selection with puromycin, transduced cells were used in experiments without cloning.

### Colony formation assay

A549- and A549-FL-shZEB1 cells (1 × 10^3^ cells/well), A549- and A549-FL-shc-Myc cells (1 × 10^3^ cells/well) and H441- and H441-FL-shc-Myc cells (4 × 10^3^ cells/well) in RPMI with 10% FBS were seeded on 6-well normal attachment plates. Plates were incubated for 9 days until colonies had formed. The wells of these plates were rinsed out by PBS, fixed with methanol, stained with Giemsa stain solution, confirmed under an inverted light microscope, and photographed.

### Pharmacological inhibition of c-Myc

We examined whether the targeting of c-Myc suppresses cell growth by the H441-FL subline using a pharmacological inhibitor. The prototype bromodomain and extra-terminal (BET) bromodomain inhibitor, JQ1, inhibits c-Myc by targeting the BRD domain of the BET family (BRD2, BRD3, BRD4, and testis-specific BRDT), which functions as an important reader molecule by associating with acetylated histones and transcription activators at specific promoter sites [16,17]. JQ1 has been reported to exhibit prominent antitumor efficacy in a subset of NSCLC cells harboring KRAS mutations through the down-regulation of Myc [16].

For Western blot analysis, the H441-FLcells were cultured under attached conditions in RPMI with 10% FBS, treated with different doses of JQ1 for 24 or 48 hours, and lysed. For cell growth assay, the H441 and H441-FL cells were cultured under attached and detached conditions in RPMI with 10% FBS, treated with different doses of JQ1 for 72 hours, and analyzed using cell count assay kit (CCK-8). The vehicle treatment (0.1% DMSO) served as a negative control.

### Statistical analysis

Data including the cell count number and drug susceptibility test results were expressed as the box-and-whisker plot (minimum, lower quartile, median, upper quartile and maximum). Statistical analyses were performed using the StatMate IV software package (ATMS, Tokyo, Japan). Statistical calculations between groups were performed using the Mann-Whitney U test (for two groups) or Kruskal-Wallis test followed by Tukey's test (for three groups). Significance was accepted at a P value less than 0.05.

## Results

### Morphological changes during long-term, low attachment cultures

**Fig 1:**
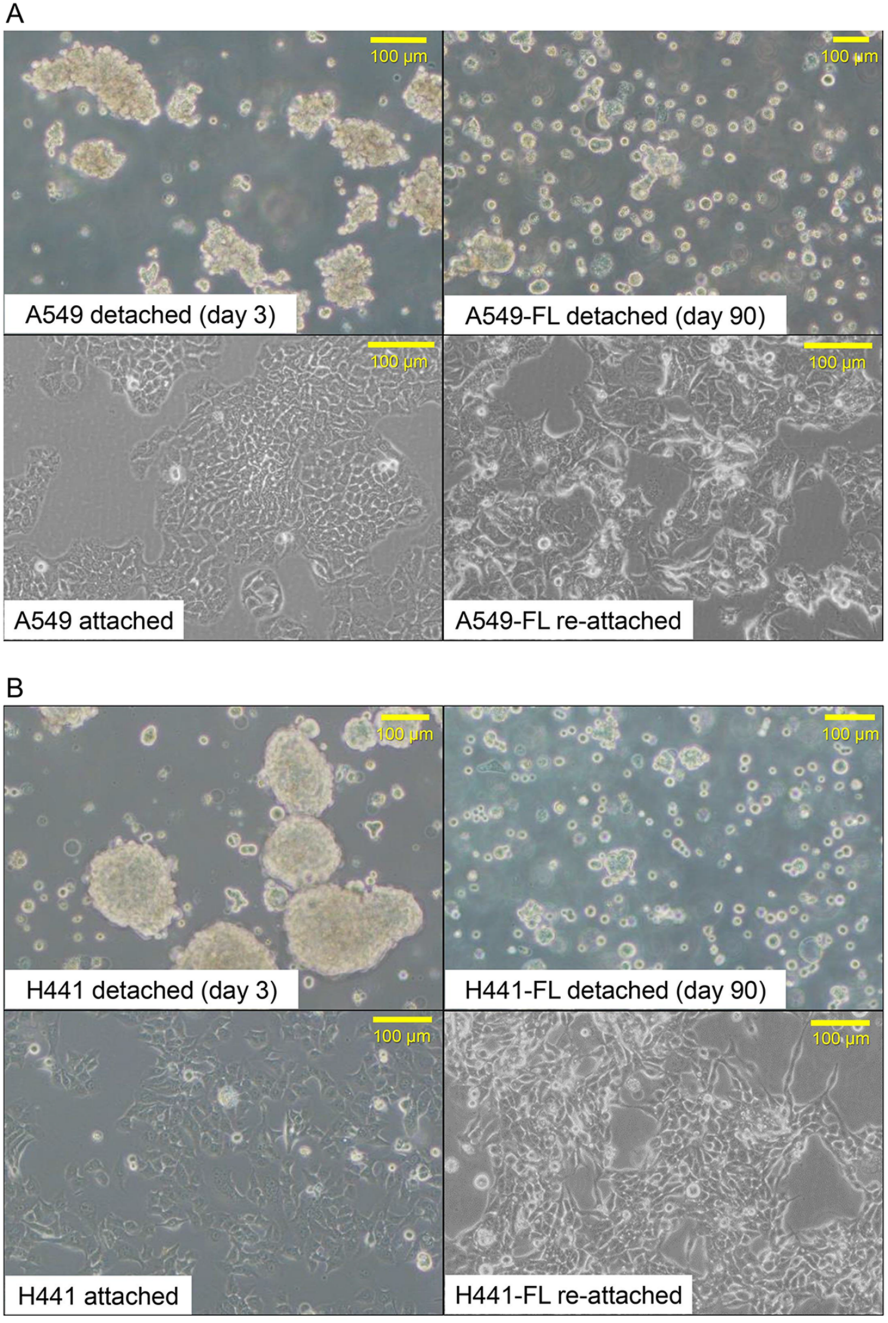
Morphological comparison of parental cell lines and FL sublines. (A) A549 parental cells showed a polygonal, epithelial morphology in attachment, and formed somewhat irregularly rounded spheroids on low-binding dishes. After long-term low attachment cultures (at day 90), cells (now designated as A549-FL) formed smaller spheroids, with some cells floating as single cells. Upon replating on ordinary dishes, A549-FL cells readily reattached and spread. The A549-FL subline, when attached on ordinary plates, showed more spindle or rounded shapes than parental cells. (B) Similar morphological changes occurred in H441 cells during long-term 3D low-attachment cultures.

When plated on low attachment cultures, A549 cells initially formed irregularly-shaped rounded spheroids, some of which clumped together to form larger clusters. H441 cells also formed more rounded, slightly smaller spheroids than those of A549 cells in the early phase of the FL culture. Chronological observations revealed that the shape of the spheroids remained stable for approximately one month, while no marked changes occurred in cell numbers or densities. After this latent period, cell clusters gradually became loose, the culture became dense and crowded, and the color of the culture medium became yellowish in a shorter time, thus requiring dividing of culture. After 3–4 months, cells appeared to have acquired the ability to stably grow at a constant rate on low-binding culture dishes. These cells, now designated as FL sublines (A549-FL and H441-FL), formed smaller spheroids than parental cells, with some cells floating as single cells, on low attachment dishes (Fig 1A and 1B). FL cells exhibited prominent plasticity with regards to cell adhesion; when plated on ordinary dishes, FL sublines readily re-attached and spread, and morphologically changed into a more spindled or rounded shape than parental cells, which displayed a polygonal, epithelial morphology (Fig 1A and 1B). These morphological changes suggested that epithelial-mesenchymal transition (EMT) occurred in FL sublines.

### Biological characterization of FL sublines

**Fig 2:**
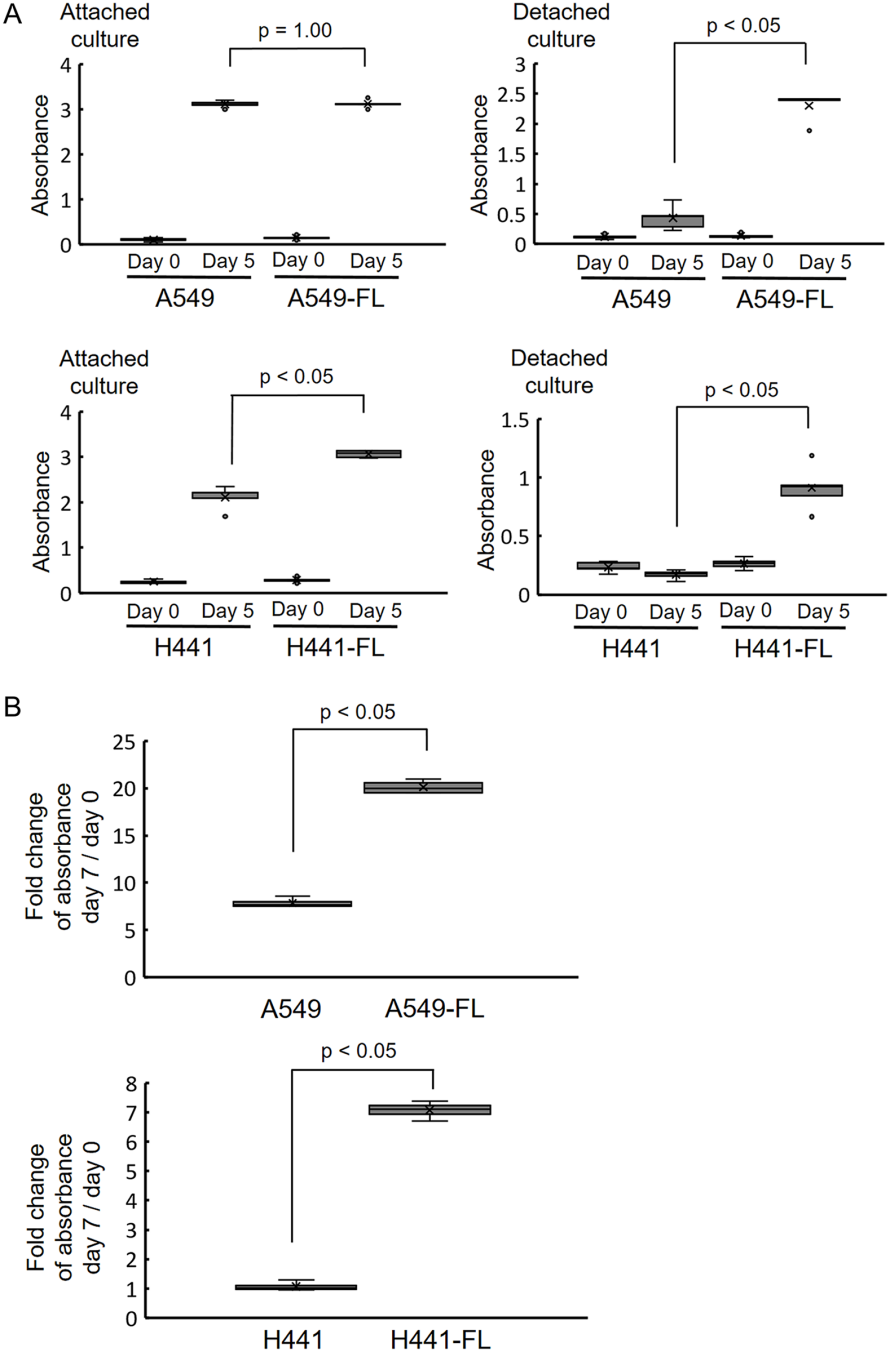
FL sublines exhibit greater cell growth potential than parental cells on low-binding cultures. (A) The A549 cell line and A549-FL subline grew at a similar rate in attachment cultures. The A549-FL subline grew at a markedly higher rate than the A549 cell line in low-attachment (detached) cultures (p < 0.05). The H441-FL subline showed a slightly higher cell growth rate than the H441 cell line in attachment cultures (p < 0.05). The H441-FL subline showed a markedly higher cell growth rate than the H441 cell line in detachment cultures (p < 0.05). Data are shown as the box-and-whisker plot (minimum, lower quartile, median, upper quartile and maximum) of 5 replicates of cultures. Statistical analysis was performed by the Mann-Whitney U test. (B) The A549-FL subline showed a markedly larger fold increase in DNA than the A549 cell line in detachment cultures (p < 0.05). The H441-FL subline also showed a markedly larger increase in DNA than the H441 cell line in detachment cultures (p < 0.05). Note that H441 cells actually showed a slight decrease in DNA during the 7 days in a detachment culture. Data are shown as the box-and-whisker plot of the ratio (day 7/day 0) of the amount of DNA extracted from 4 samples of replicates of cultures. Statistical analysis was performed by the Mann-Whitney U test.

We found that FL sublines showed stronger cell growth abilities than parental cell lines in low attachment cultures. As shown in Fig 2A, the A549 cell line and A549-FL subline grew at similar rates in attachment cultures. In contrast, in low-attachment (detached) cultures, the A549-FL subline grew at a markedly higher rate than the parental A549 cell line (Fig 2A). The H441-FL subline grew at a slightly higher rate than the H441 cell line in attachment cultures (Fig 2A), whereas in detachment cultures, the H441-FL subline showed a markedly higher cell growth rate than the H441 cell line (Fig 2A). Cell numbers in the parental H441 cell line appeared to be slightly lower on day 5 under detached conditions.

In order to confirm the data obtained by the colorimetric cell count assay, we measured DNA extracted on days 0 and 7 in the detachment culture, and investigated whether increases in absorbance reflected greater DNA synthesis. As shown in Fig 2B, FL sublines showed a markedly larger fold increase in DNA during the 7-day culture period than the parental cell line in low attachment cultures.

**Fig 3:**
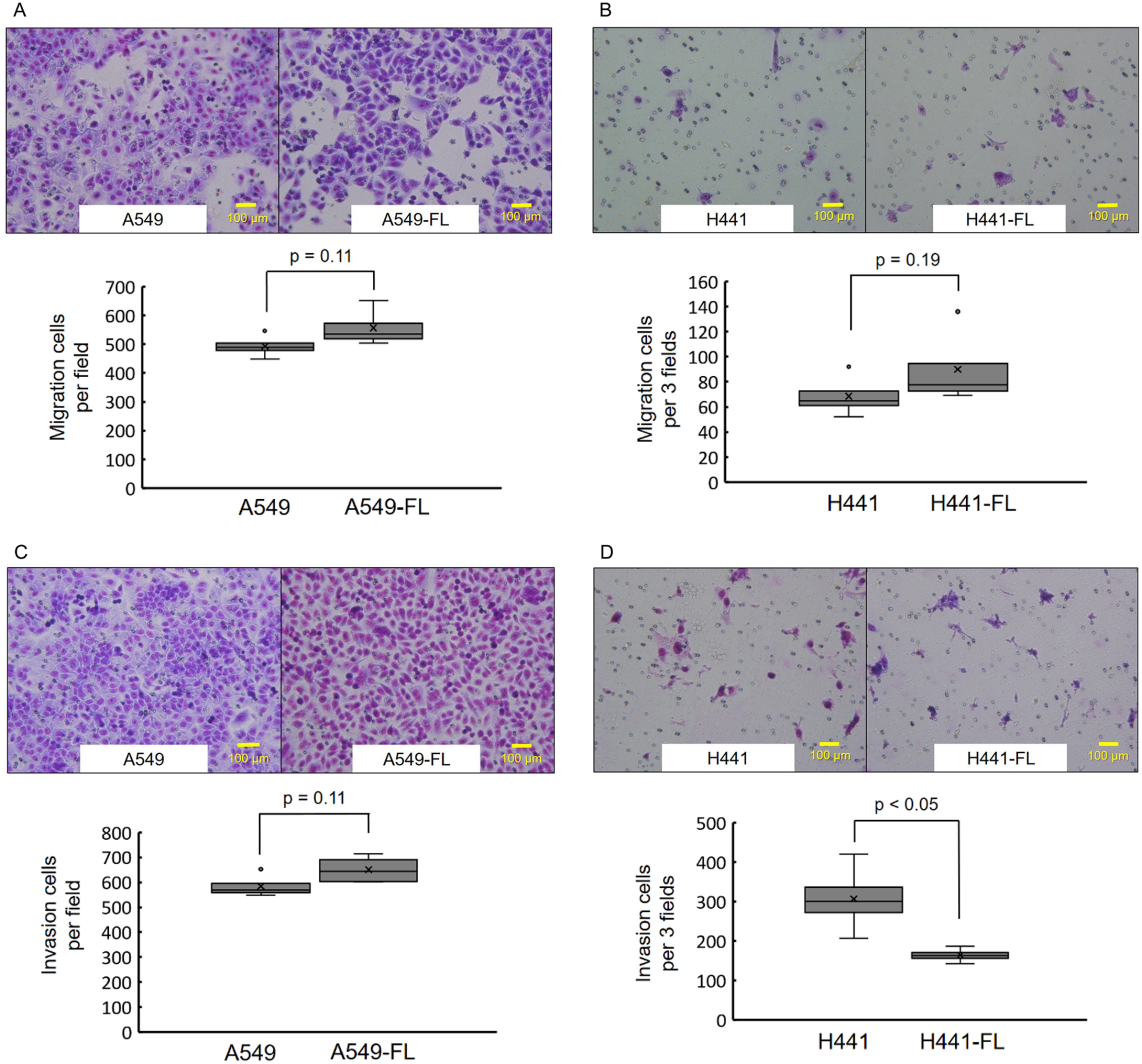
Transwell migration and invasion assays. (A, B) Transwell migration assay. Representative microscopic images of cells that migrated through the transwell in the migration assay. (Giemsa stain, magnification ×200). No significant differences were observed between the FL subline and parental cell line for A549 or H441 cells. The quantitation of cells that migrated through the transwell in the migration assay. The data are shown as the box-and-whisker plot of the mean number of migrated cells per visual field (A) or 3 visual field (B) (magnification ×100) of 4 replicate wells. No significant differences were found between the FL subline and parental cell line for A549 cells or for H441 cells. (C, D) Transwell Matrigel invasion assay. Representative microscopic images of cells that invaded through the transwell in the Matrigel invasion assay. (Giemsa stain, magnification ×200). No significant differences were observed between the FL subline and parental cell line for A549 or H441 cells. Quantitation of cells that invaded through the transwell in the Matrigel invasion assay. The data are shown as the box-and-whisker plot of the mean number of cells per visual field (C) or 3 visual field (D) (magnification ×100) of 4 replicate wells. The results obtained were similar between A549 and A549-FL cells. Invasion was slightly weaker in H441-FL cells than in H441 cells. Statistical analysis was performed by the Mann-Whitney U test.

We then examined cell migration and invasion in parental and FL cells using a transwell assay. The results obtained are shown in Fig 3A–3D. No significant differences were observed in cell migration between FL sublines and parental cells (Fig 3A and 3B). Similarly, no significant differences were observed in invasion between A549 and A549-FL cells (Fig 3C). Invasion was slightly weaker in H441-FL cells than in H441 cells (Fig 3D).

**Fig 4:**
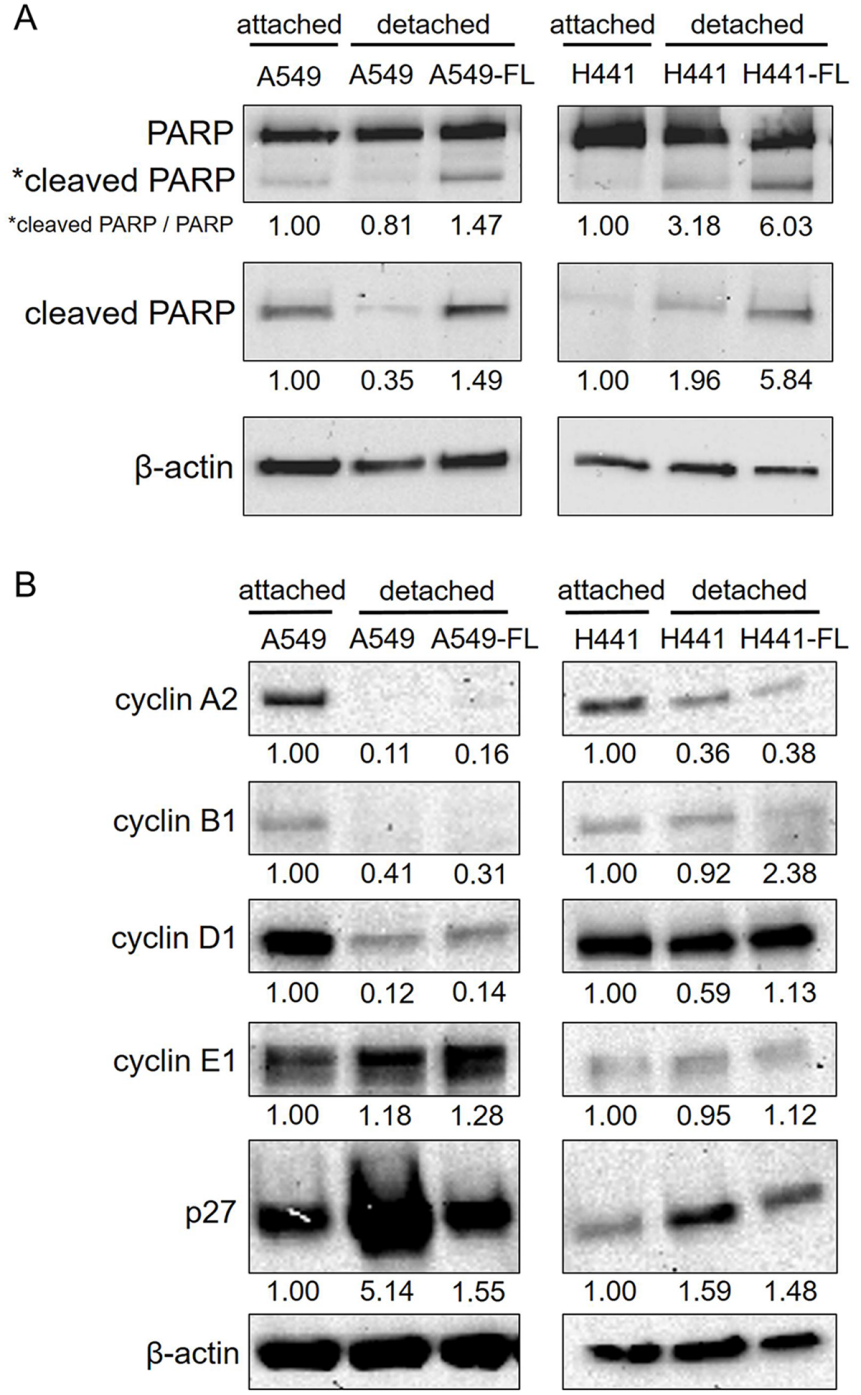
Levels of apoptosis and cell cycle regulators in parental and FL cells in detachment. (A) Parental and FL cells in low attachment cultures were lysed and subjected to a Western blot analysis with antibodies to poly ADP-ribose polymerase (PARP), cleaved PARP, and β-actin. Parental cells in attachment cultures were included for comparison. Under detached conditions, apoptosis was greater in FL cells than in parental cells. * in PARP indicates the cleaved fragment of PARP. (B) Parental and FL cells in low attachment cultures were lysed and subjected to a Western blot analysis with antibodies to cyclin A2, B1, D1, E1, p27, and β-actin. While the levels of cyclin A2, B1, D1, and E1 were similar between parental A549 and A549-FL cells, p27 levels were markedly lower in A549-FL cells than in parental A549 cells. Similarly, H441-FL cells showed reduced p27 levels. Number below gel images represent the relative protein levels of the indicated proteins after normalization to β-actin. Data shown are representative of 2 independent experiments.

The high cell growth rate of FL cells under detached conditions may reflect high cell proliferation (cell division) and/or low apoptotic rates in detachment. Therefore, we measured apoptosis levels in parental and FL cells cultured under detached conditions using a Western blot analysis of the cleaved fragment of PARP. Prior to the experiment, we speculated that FL cells may have acquired the ability to suppress anoikis, i.e., apoptosis caused by the loss of cell-substratum adhesion. The results obtained are shown in Fig 4A. We found higher levels of cleaved PARP in FL sublines than in parental cells under detached conditions.

The above results indicate that the increased cell growth rate of FL cells under detached conditions is attributable to a high rate of cell proliferation that exceeded the elevated rate of apoptosis. In order to elucidate the underlying molecular changes, we examined the expression levels of the cell cycle regulators, cyclin A2, B1, D1, E1, and the cyclin-dependent kinase (cdk) inhibitor p27 in parental cells in attachment cultures, and in parental and FL cells in detachment cultures (Fig 4B). The levels of cyclin A2, B1, and D1 were markedly lower, whereas those of p27 were higher in parental A549 cells following detachment than in A549 cells in attachment cultures. These changes were consistent with the slower cell growth of parental A549 cells under detached than attached conditions. We then compared the levels of cyclins and p27 between parental and FL cells under detached conditions. While the levels of cyclin A2, B1, D1, and E1 were similar between parental and FL cells, p27 levels in FL cells decreased to a level that was similar to that in parental cells in attachment cultures. Parallel experiments with H441 parental and FL cells showed similar results for p27, whereas the levels of most cyclins remained unchanged, except for cyclin A2, which decreased in detached cells. We also examined the levels of p21 and p16; however, their expression levels were undetectably low in all samples tested (data not shown).

### Murine metastasis models of FL sublines representing high metastatic potential

**Fig 5:**
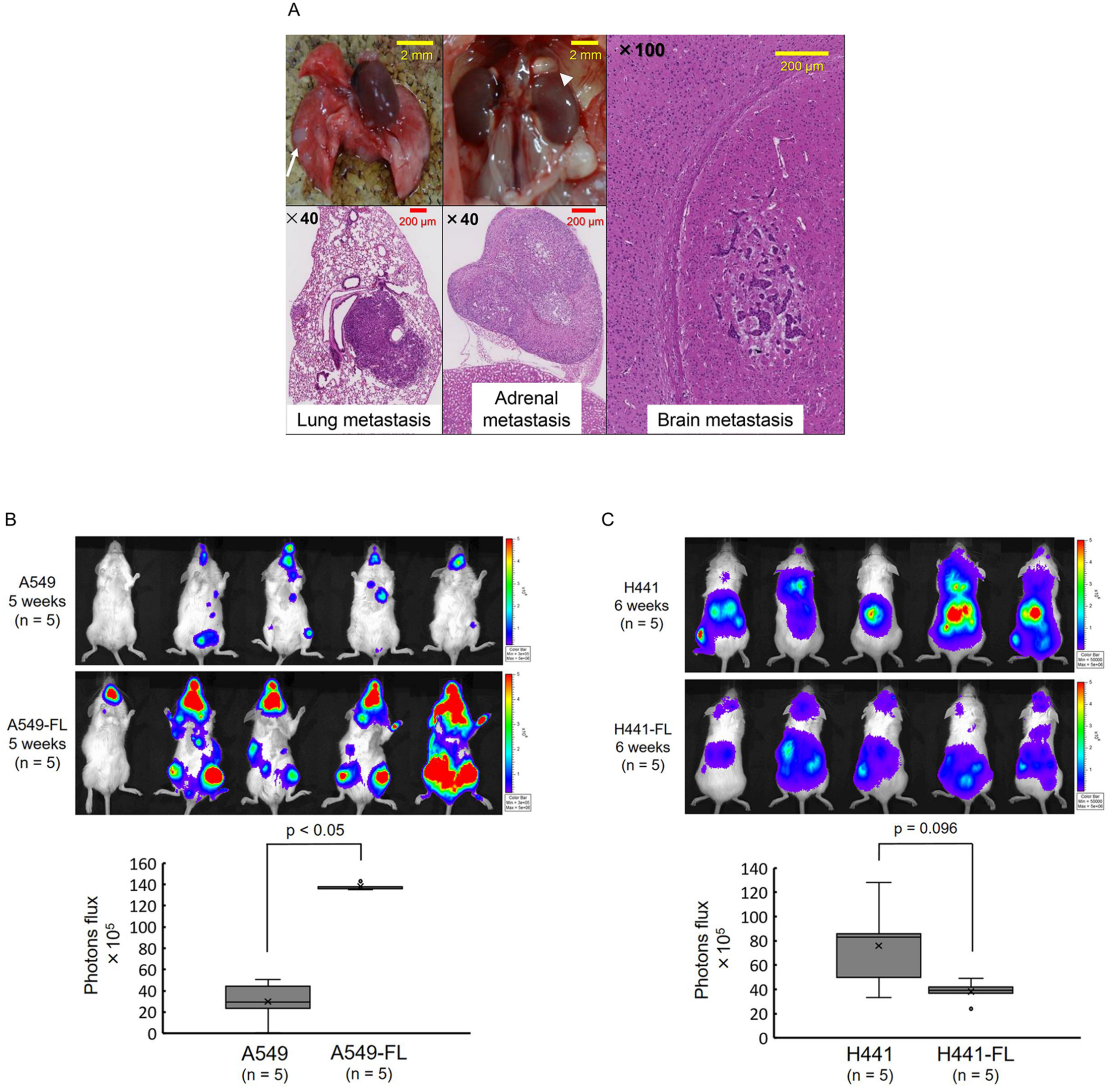
Metastatic tumor formation after an intracardiac injection of parental and FL cells. (A) Metastatic tumors were formed in multiple organs in all mice injected with parental or FL cells. Representative macroscopic and microscopic pictures are shown of lung (white arrow), adrenal (white arrow head), and brain metastases of luc-A549 cell lines. (B) *In vivo* luciferase imaging of luc-A549 cell lines and luc-A549-FL sublines at 5 weeks post-implantation. Representative images from ventral view are shown. Quantification of tumor-derived photons at 5 weeks post-implantation. Data are shown as the mean ± standard deviation of the photon flux of 5 animals. The Luc-A549-FL subline showed markedly greater metastatic tumor growth than luc-A549 cells. (C) *In vivo* luciferase imaging of luc-H441 cell lines and luc-H441-FL sublines at 6 weeks post-implantation. Representative images from dorsal view are shown. Quantification of tumor-derived photons at 6 weeks post-implantation. No significant differences were observed in tumor-derived photons between luc-H441 and luc-H441-FL cells. Data are shown as the box-and-whisker plot of the photon flux of 5 animals. Statistical analysis was performed by the Mann-Whitney U test.

We investigated the metastatic potentials of A549 and H441 parental cell lines and their FL sublines following an intracardiac injection into NOD/SCID mice. In cell lines and their FL sublines, we reproducibly produced multiple organ metastasis *in vivo* (Fig 5A). We evaluated metastasis using two methods: 1) a quantitative analysis of the tumor-derived photons of luciferase-based bioluminescence, and 2) semi-quantitative histopathological scoring. The bioluminescence imaging of murine models indicated that organ metastasis was greater in the luc-A549-FL subline than in luc-A549 cells (Fig 5B). Among mice injected with luc-A549-FL cells, mice had to be sacrificed 1 week earlier on average than those injected with luc-A549 parental cell lines due to an increased tumor burden and loss of body weight (S2 Fig). A histopathological analysis of the harvested organs revealed no significant differences between parental luc-A549 cells and luc-A549-FL sublines (Table 2A); however, this discrepancy may be attributable to the different time points at which organs were harvested in luc-A549 and luc-A549-FL cells.

**Table 2:** Comparison of murine metastasis models with a semi-quantitative evaluation. A, A549 vs A549-FL; B, H441 vs H441-FL.

**A**													
Cell line	A549						A549-FL						
Model number	1	2	3	4	5	total	1	2	3	4	5	total	p-value
Brain		2	3	3	2	10	2	2	3	2	3	12	N.S.
Teeth		3	3	3	3	12		3	3	3	3	12	N.S.
Lungs	3	2		3		8		2				2	N.S.
Stomach		2				2						0	N.S.
Liver		1		2		3						0	N.S.
Pancreas		3				3				2	2	4	N.S.
Spleen						0						0	N.S.
Kidneys		1	2	2	1	6		2	2	2	2	8	N.S.
Adrenal gland		3	3	2	3	11	3	3	3	3	3	15	N.S.
Ovaries		3	2	3	3	11	3	3	3	3	3	15	N.S.
Bowels				2		2	3					3	N.S.
Spine		3	1		3	7		2	2	3	3	10	N.S.
Femurs		3	3			6		2	3	3	3	11	N.S.
Number of involved organs	1	11	7	8	6	36	4	8	7	8	8	35	0.842
Total score	3	26	17	20	15	81	11	19	19	21	22	92	0.690
**B**													
Cell line	H441						H441-FL						
Model number	1	2	3	4	5	total	1	2	3	4	5	total	p-value
Brain			2	1	2	5	4	4	4	2	3	17	< 0.05
Teeth						0	3	3	3		3	12	< 0.05
Lungs					2	2			1			1	N.S.
Stomach						0						0	N.S.
Liver						0			2			2	N.S.
Pancreas						0	2	2			2	6	N.S.
Spleen						0						0	N.S.
Kidneys						0						0	N.S.
Adrenal gland	3	3	3	3	3	15	3	3	3	3	3	15	N.S.
Ovaries	3			3	3	9	3	3	3	3	3	15	N.S.
Bowels						0						0	N.S.
Spine	2	2			2	6		2		3	3	8	N.S.
Femurs					3	3				3		3	N.S.
Number of involved organs	3	2	2	3	6	16	5	6	6	5	6	28	0.056
Total score	8	5	5	7	15	40	15	17	16	14	17	79	< 0.05

We also made similar comparisons for the luc-H441 parental cell line and luc-H441-FL sublines. As shown in Fig 5C, tumor-derived photons showed similar intensities between luc-H441 and luc-H441-FL cells. In some mice, luc-H441 cells appeared to have slightly more photons than luc-H441-FL cells (Fig 5C). However, a semi-quantitative histopathological evaluation showed that organ metastasis was greater in the luc-H441-FL subline than in the parental luc-H441 cell line (Table 2B). Luc-H441-FL cells displayed a clear increase in metastasis to the brain and teeth.

### Gene expression profiling and analysis of FL sublines revealed the expression of EMT-related factors

**Fig 6:**
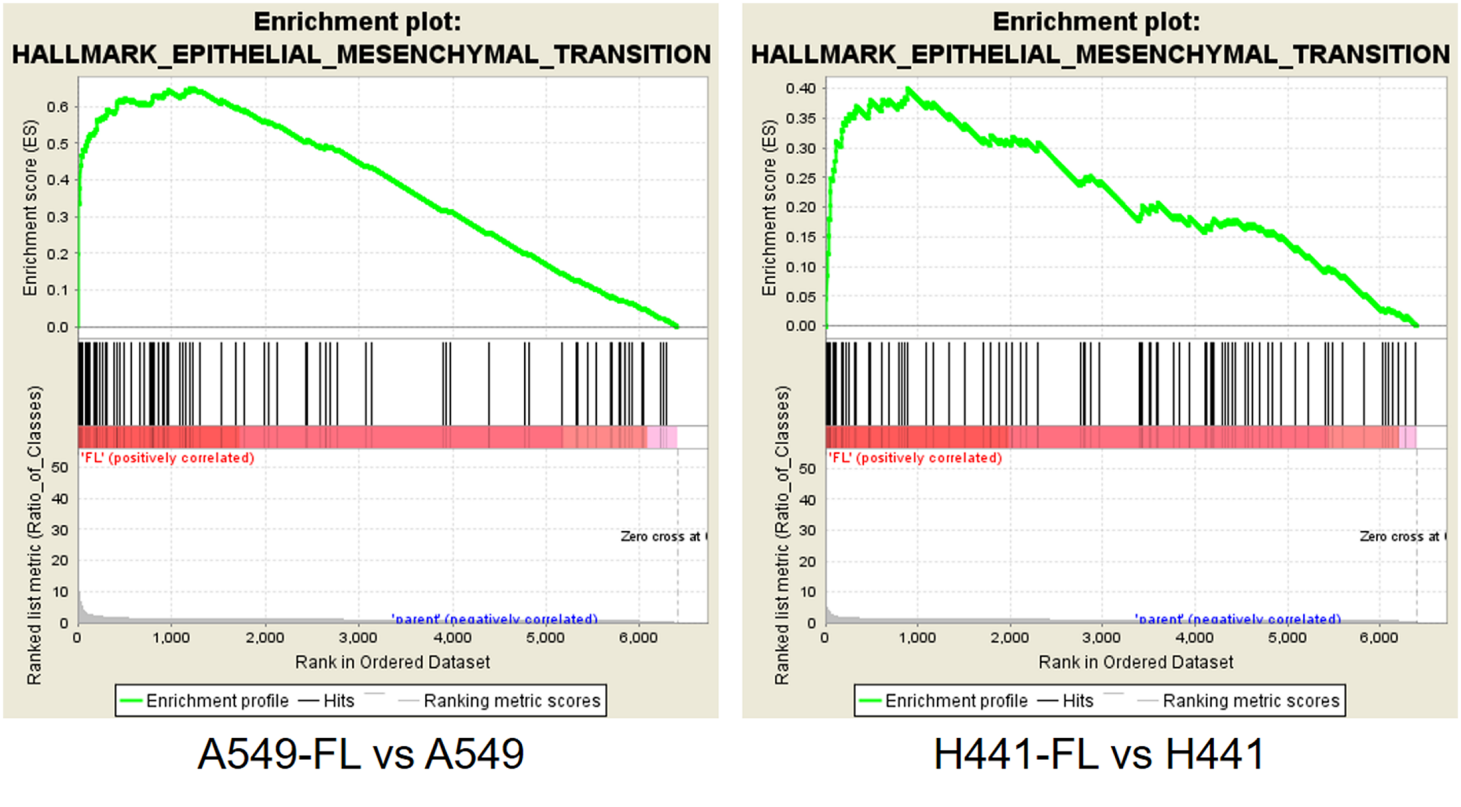
Selected examples of a gene set enrichment analysis (GSEA). Among the “hallmark gene sets” analyzed, EPITHELIAL MESENCHYMAL TRANSITION (EMT) was noted in A549-FL and H441-FL cells versus their parental counterparts. Enrichment plots for EMT signatures (A549-FL vs A549 and H441-FL vs H441) are shown.

In an attempt to elucidate the molecular mechanisms responsible for increased cell growth in suspension cultures and the higher metastatic potential of FL sublines *in vivo*, we performed a gene expression profiling analysis. Using the criteria described in Table 3A (fold change < -3.0 or > +3.0, and signal level >500), we identified 657 differentially expressed genes between A549 and A549-FL cells. These included 302 up-regulated and 355 down-regulated genes in A549-FL versus A549 cells. (Table 3A). Using the same criteria, we detected 325 differentially expressed genes between H441 and H441-FL cells. These included 168 up-regulated and 157 down-regulated genes in H441-FL versus H441 cells (Table 3A). Since a functional analysis of the individual genes listed in Table 3A was time-consuming and labor intensive, we decided to use the GSEA (gene set enrichment analysis) method [18]. GSEA based on hallmark gene sets revealed 12 and 9 gene sets, respectively, enriched in the A549-FL and H441-FL sublines from their parental counterparts (Table 3B and 3C). Of these gene sets, six gene sets, namely, EPITHELIAL MESENCHYMAL TRANSITION (EMT), TNFA SIGNALING VIA NFKB, KRAS SIGNALING UP, IL6 JAK STAT3 SIGNALING, HYPOXIA, and ESTROGEN RESPONSE LATE were common in A549-FL and H441-FL cells (Fig 6 and Table 3B and 3C). Of these, EMT was an overwhelmingly enriched gene set in A549-FL cells, and ranked relatively high in H441-FL cells.

**Table 3:** A. Number of gene alternations in the gene expression array with limiting criteria in the FL subline (versus the parental cell line). B. Gene set enrichment analysis: significantly enriched in the A549-FL subline (versus the A549 cell line). C. Gene set enrichment analysis: significantly enriched in the NCI-H441-FL subline (versus the NCI-H441 cell line).

**A**		
Cell line	Up-regulation	Down-regulation
A549-FL vs A549	302	355
H441-FL vs H441	168	157
**Limiting criteria**		
1. Expression signal ratio; fold change > +3.0 or < -3.0		
2. Signal level > 500		
3. NM-gene in description notation (GenBank)		
**B**		
Hallmark gene set	p value	FDR q-val
EPITHELIAL MESENCHYMAL TRANSITION	0.00000	0.00000
ANGIOGENESIS	0.00229	0.00452
TNFA SIGNALING VIA NFKB	0.00000	0.02924
KRAS SIGNALING UP	0.00201	0.05224
ESTROGEN RESPONSE LATE	0.02305	0.12205
HEME METABOLISM	0.01108	0.12263
COAGULATION	0.03252	0.12691
P53 PATHWAY	0.03528	0.12941
APOPTOSIS	0.01705	0.14148
IL6 JAK STAT3 SIGNALING	0.03661	0.14352
HYPOXIA	0.01602	0.15051
INTERFERON GAMMA RESPONSE	0.04614	0.17355
**C**		
Hallmark gene set	p value	FDR q-val
EPITHELIAL MESENCHYMAL TRANSITION	0.00000	0.00000
ANGIOGENESIS	0.00229	0.00452
TNFA SIGNALING VIA NFKB	0.00000	0.02924
KRAS SIGNALING UP	0.00201	0.05224
ESTROGEN RESPONSE LATE	0.02305	0.12205
HEME METABOLISM	0.01108	0.12263
COAGULATION	0.03252	0.12691
P53 PATHWAY	0.03528	0.12941
APOPTOSIS	0.01705	0.14148
IL6 JAK STAT3 SIGNALING	0.03661	0.14352
HYPOXIA	0.01602	0.15051
INTERFERON GAMMA RESPONSE	0.04614	0.17355

FDR = false discovery rate

### Long-term low attachment cultures induced EMT in the A549 cell line

**Fig 7:**
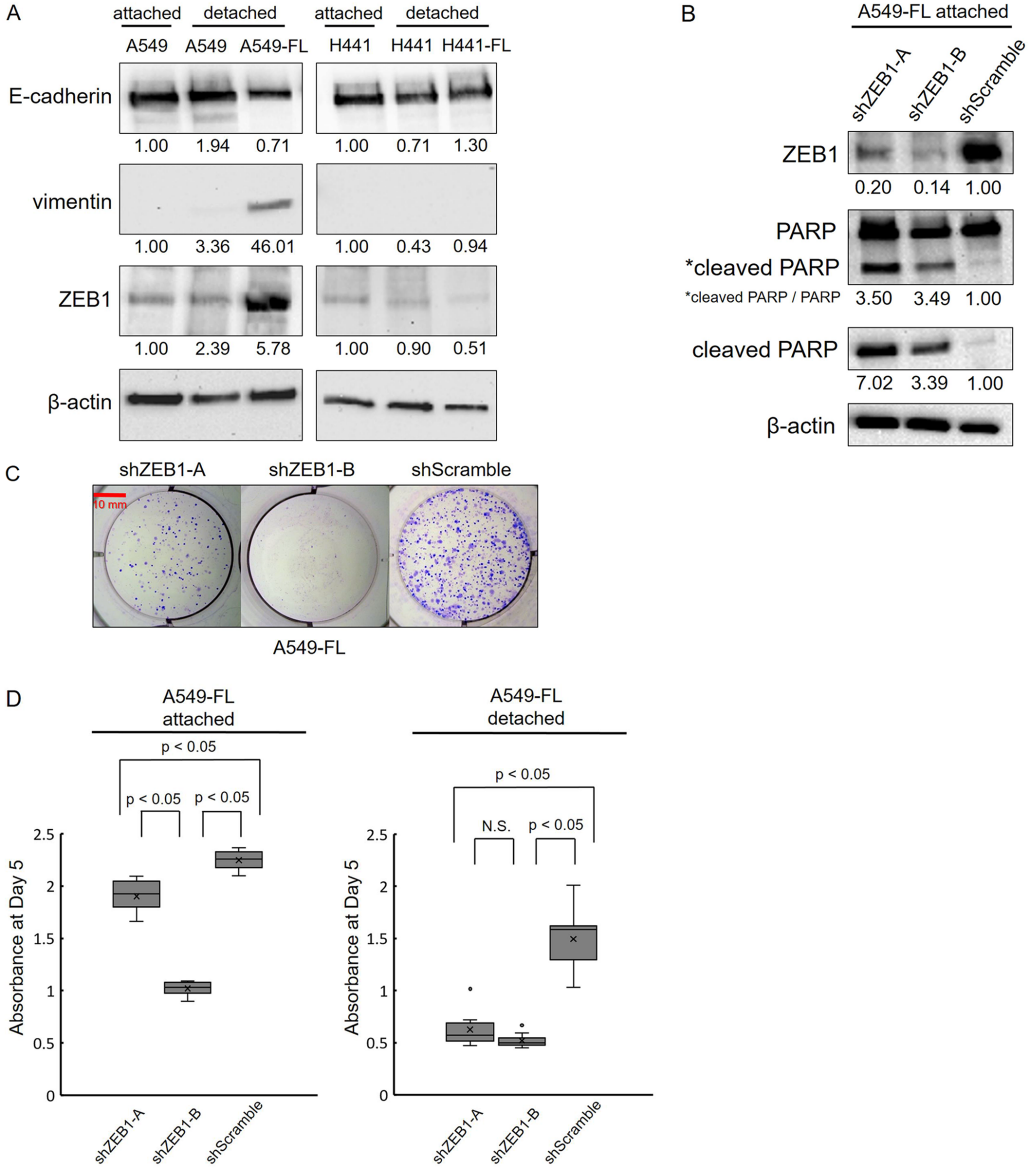
Zinc finger E-box binding homeobox 1 (ZEB1) was induced in A549-FL cells and its knockdown (KD) led to apoptosis and the inhibition of cell growth in A549-FL cells. (A) Parental cells under detached conditions (3 days after detachment) and FL cells in a continued detachment (low attachment) culture were lysed and subjected to a Western blot analysis with antibodies to E-cadherin, vimentin, ZEB1, and β-actin. A549 cells in an attachment culture were included for comparison. Long-term low attachment (detached) cultures caused the slightly weaker expression of E-cadherin and stronger expression of vimentin and ZEB1 in A549-FL cells than in A549 cells. (B-D) A549-FL cells were transduced with lentiviral vectors (shZEB1-A, shZEB1-B, and shScramble), and after a brief selection with puromycin, used for a Western blot analysis, colony formation assay, and cell count assay, without cloning (see the Materials and methods for details). (B) ZEB1 KD induced apoptosis in A549-FL cells, as indicated by the emergence of the cleaved PARP fragment. Number below gel images represent the relative protein levels of the indicated proteins after normalization to β-actin. Data shown are representative of 2 independent experiments. (C) Representative images of macroscopic colony formation assay of 4 replicates of cultures (Giemsa stain). Colony formation was inhibited more by shZEB1-A and shZEB1-B than by the control (shScramble). (D) Cell growth was inhibited more by shZEB1-A and shZEB1-B than by the control (shScramble) in attachment and detachment cultures. Data are shown as the box-and-whisker plot of 10 replicates. Statistical analysis was performed by the Kruskal-Wallis test followed by Tukey's test.

In order to confirm whether EMT had occurred in the A549-FL subline, we assessed the protein levels of E-cadherin, vimentin, and ZEB1 using a Western blot analysis. The weak expression of E-cadherin and strong expression of vimentin are traditional markers of EMT and these expression patterns are currently used to identify cells that have undergone EMT in circulating tumor cells [19]. ZEB1 functions as a master regulator of epithelial plasticity in cancer cell invasion and human tumor progression *in vivo* [20]. The results of Western blots are shown in Fig 7A. Long-term detachment induced the slightly weaker expression of E-cadherin and the stronger expression of vimentin and ZEB1 in A549-FL than in A549 cells under detached conditions. Similar results were obtained when A549 and A549-FL cells were compared under attached conditions (data not shown). Parallel experiments with H441 and H441-FL cells showed no clear evidence of EMT in H441-FL cells.

### Effects of ZEB1 KD in A549-FL cells

Since ZEB1 is a master regulator of EMT, we hypothesized that ZEB1 contributes to the enhanced growth of A549-FL cells under detached conditions. In order to investigate this, we examined the effects of ZEB1 KD in A549-FL cells. We used two ZEB1 KD vectors (shZEB1-A and shZEB1-B) that targeted different regions of ZEB1 mRNA. As shown in Fig 7B, both sh-RNA vectors effectively down-regulated ZEB1 protein levels. ZEB1 KD induced apoptosis in A549-FL, as shown by the appearance of cleaved PARP in Western blots (Fig 7B). Colony formation was also markedly inhibited by ZEB1 KD (Fig 7C). Furthermore, cell growth was strongly inhibited by ZEB1 KD under attached and detached conditions (Fig 7D). We tested the effects of ZEB1 KD in parental A549 cells as well. As shown in S4 Fig, colony formation was modestly inhibited by ZEB1 KD in A549, but the inhibitory effect of ZEB1 KD was not as strong as in A549-FL. Similarly, cell growth was modestly inhibited by one of the two ZEB1 KD vectors, sh-ZEB1-B, but not by sh-ZEB1-A. In detached condition, in particular, sh-ZEB1-A vector showed a paradoxical stimulating effect in parental A549 cells. These differences may reflect inefficiency of ZEB1 KD and/or off-target effect of sh-ZEB1-A in this particular setting.

### Identification of c-Myc amplification in the H441-FL subline

**Fig 8:**
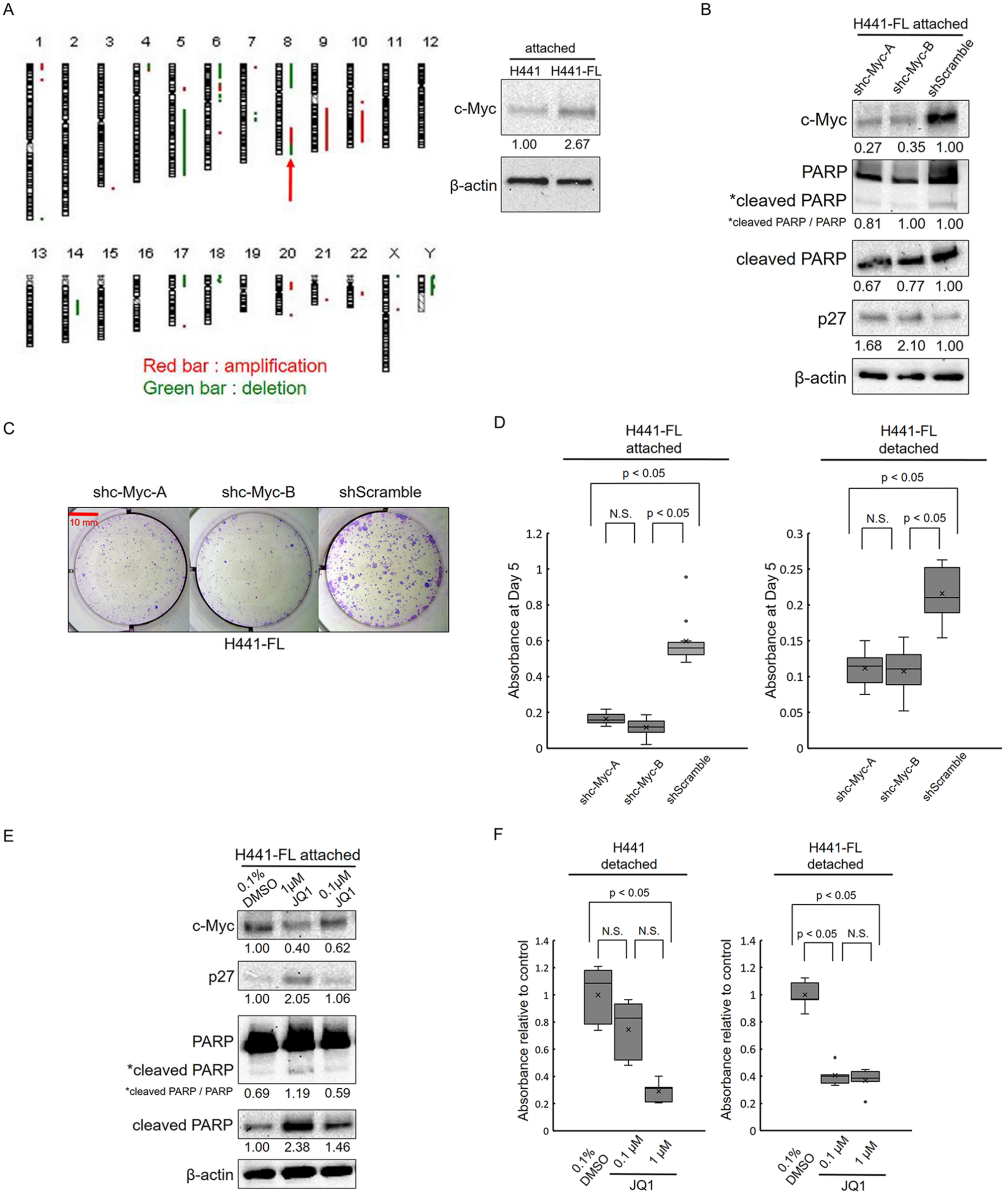
c-Myc was amplified in H441-FL cells and its inhibition decreased the growth of these cells. (A) A copy number analysis by CGH demonstrated genomic regions of amplification (red bar) and deletion (green bar) in H441-FL cells. The red arrow indicates amplification on chromosome 8q24, which harbors the c-Myc gene. c-Myc expression was increased in H441-FL sublines at the protein level. (B-D) H441-FL cells were transduced with lentiviral vectors (shc-Myc-A, shc-Myc-B, and shScramble), and after a brief selection with puromycin, used for a Western blot analysis, colony formation assay, and cell count assay, without cloning (see the Materials and methods for details). (B) C-Myc KD did not induce apoptosis, but caused a modest increase in p27 levels. (C) Representative images of macroscopic colony formation assay of 4 replicates of cultures (Giemsa stain). Colony formation was inhibited more by shc-Myc-A and shc-Myc-B than by the control (shScramble). (D) Cell growth was inhibited more by shc-Myc-A and shc-Myc-B than by the control (shScramble) in attachment and detachment cultures. Data are shown as the box-and-whisker plot of 10 replicates. Statistical analysis was performed by the Kruskal-Wallis test followed by Tukey's test. (E) The H441 FL subline was treated with the indicated doses of JQ1 for 24 or 48 hours, or with 0.1% DMSO as a negative control. Cell lysates were subjected to a Western blot analysis for c-Myc, p27, PARP, cleaved PARP, and β-actin. An analysis of c-Myc was performed after a 24-hour treatment, while that of the others was performed after a 48-hour treatment. The results obtained demonstrated that JQ1 caused the depletion of c-Myc, an increase in p27, and the induction of cleaved PARP in a dose-dependent manner. (F) The JQ1 treatment inhibited the growth of H441 and H441-FL cells in detachment cultures. The H441-FL subline was slightly more sensitive to the treatment with 0.1 μM JQ1 than the parental H441 cell line. Data are shown as the box-and-whisker plot of 5 replicates. Statistical analysis was performed by the Kruskal-Wallis test followed by Tukey's test. (A, B, E) Number below gel images represent the relative protein levels of the indicated proteins after normalization to β-actin. Data shown are representative of 2 independent experiments.

The H441-FL subline did not show any evidence of EMT. In a search for other candidate molecules, we performed a copy number analysis using CGH. The CGH analysis of H441 and H441-FL cells revealed genomic regions at which copy number differences were detected between H441-FL sublines and H441 parental cell lines. Eighty-four copy number alternations, including 29 amplifications and 55 deletions, were detected between H441-FL and H441 (Table 4). We focused on the c-MYC gene at 8q-24.21 (Fig 8A and Table 4), which showed 2.8 to 3.7-fold amplifications in H441-FL cells versus H441 cells. A Western blot analysis also confirmed that the expression of c-Myc was stronger at the protein level in H441-FL versus H441 parental cells (Fig 8A). We checked expression levels of c-Myc in A549 and A549-FL, but the expression of c-Myc was only slightly increased in A549-FL vs. A549 cells (S5 Fig).

**Table 4:** Significantly amplified gene lesions with gene names in a copy number analysis of the NCI-H441-FL subline (versus the NCI-H441 cell line).

No	Chr	Cytoband	Start	Stop	Amplification*	p-value	Gene Names
1	1	p36.33—p36.21	564424	12912625	0.422733	0	MIR1977, OR4F29, OR4F3, OR4F16, LOC100133331, NCRNA00115, LOC643837, FAM41C, FLJ39609, SAMD11
2	1	p36.11	24610598	25113062	0.309153	2.18E-23	GRHL3, C1orf201, NIPAL3, RCAN3, C1orf130, SRRM1, CLIC4
3	3	q29	1.96E+08	1.97E+08	0.256528	1.24E-23	FBXO45, LRRC33, C3orf34, PIGX, PAK2, SENP5, NCBP2, LOC152217, PIGZ, MFI2
4	4	p16.1	9766686	10337296	0.600216	1.92E-32	DRD5, SLC2A9, WDR1
5	5	p13.1—p11	39200053	46100367	0.281653	5.72E-144	FYB, C9, DAB2, PTGER4, TTC33, PRKAA1, RPL37, SNORD72, CARD6, C7
6	6	p21.31—p21.1	35434789	45332930	0.57272	0	FANCE, RPL10A, TEAD3, TULP1, FKBP5, LOC285847, C6orf81, C6orf126, C6orf127, CLPS
7	6	p21.31	35434789	36386931	2.478773	0	FANCE, RPL10A, TEAD3, TULP1, FKBP5, LOC285847, C6orf81, C6orf126, C6orf127, CLPS
8	6	q21	1.09E+08	1.09E+08	0.408493	3.07E-15	FOXO3
9	7	p22.1	4527869	4810270	0.301427	7.34E-13	FOXK1
10	8	q22.3—q24.21	1.03E+08	1.29E+08	0.860969	0	NCALD, RRM2B, UBR5, ODF1, KLF10, AZIN1, ATP6V1C1, C8orf56, BAALC, FZD6
11	8	q22.3—q23.3	1.03E+08	1.18E+08	0.559988	0	NCALD, RRM2B, UBR5, ODF1, KLF10, AZIN1, ATP6V1C1, C8orf56, BAALC, FZD6
12	8	q23.3—q24.12	1.18E+08	1.2E+08	1.267073	2.36E-79	EIF3H, UTP23, RAD21, C8orf85, SLC30A8, MED30, EXT1, SAMD12
13	8	q24.12	1.2E+08	1.22E+08	2.270912	0	SAMD12, TNFRSF11B, COLEC10, MAL2, NOV, ENPP2, TAF2, DSCC1, DEPDC6, COL14A
14	8	q24.12	1.2E+08	1.2E+08	1.659477	3.46E-13	SAMD12
15	8	q24.12	1.21E+08	1.22E+08	3.088803	2.62E-38	COL14A1, MRPL13, MTBP
16	8	q24.13	1.23E+08	1.23E+08	2.988724	8.19E-52	
17	8	q24.13—q24.21	1.26E+08	1.29E+08	1.529374	5.92E-304	ZNF572, SQLE, KIAA0196, NSMCE2, TRIB1, FAM84B, POU5F1B, LOC727677, **MYC**, PVT1
18	8	q24.21	1.29E+08	1.29E+08	1.882927	9.64E-27	**MYC**, PVT1, MIR1204, MIR1205, MIR1206, MIR1207, MIR1208
19	9	q21.11—q34.3	70984481	1.41E+08	0.542504	0	PGM5, C9orf71, PIP5K1B, FAM122A, PRKACG, FXN, TJP2, FAM189A2, APBA1, PTAR1
20	10	q21.2	61424071	61562752	0.474049	3.34E-14	SLC16A9, CCDC6
21	10	q22.2—q26.13	75178439	1.27E+08	0.546666	0	ZMYND17, PPP3CB, USP54, MYOZ1, SYNPO2L, AGAP5, BMS1P4, SEC24C, FUT11, CHCHD1
22	17	q25.3	79438588	80137769	0.275018	3.10E-19	ACTG1, FSCN2, C17orf70, NPLOC4, TSPAN10, PDE6G, C17orf90, CCDC137, ARL16, HGS
23	20	p12.2—p11.1	11774340	26075841	0.525664	0	BTBD3, SPTLC3, ISM1, TASP1, ESF1, C20orf7, SEL1L2, MACROD2, FLRT3, KIF16B
24	20	p12.2—p12.1	11851056	13653845	0.844292	2.58E-41	BTBD3, SPTLC3, ISM1, TASP1
25	20	p11.23—p11.1	18669289	26075841	0.39026	3.93E-39	DTD1, HSPC072, LOC100270804, C20orf79, SLC24A3, LOC100130264, RIN2, NAA20, CRNKL1, C20orf26
26	20	q13.33	62163415	62447995	0.321891	2.19E-13	PTK6, SRMS, C20orf195, PRIC285, GMEB2, STMN3, RTEL1, TNFRSF6B, ARFRP1, ZGPAT
27	21	q22.13	38569806	39136373	0.347032	4.03E-30	TTC3, DSCR9, DSCR3, DYRK1A, KCNJ6
28	22	q12.1—q12.3	26877968	33743143	0.373809	0	HPS4, SRRD, TFIP11, TPST2, MIR548J, CRYBB1, CRYBA4, MIAT, MN1, PITPNB
29	X	p11.22	53742417	53822101	0.574541	2.94E-11	

* Fold changes (H441-FL vs. H441) expressed as log_2_ values.

### Effects of c-Myc KD and pharmacological inhibition

In order to examine the significance of c-Myc amplification, we knocked down c-Myc using two lentiviral shRNA vectors (shc-Myc-A and shc-Myc-B) targeting different regions of c-Myc mRNA. We confirmed that both shRNA vectors down-regulated c-Myc at the protein level in H441-FL cells (Fig 8B). Unlike ZEB1 KD, no significant changes were observed in apoptosis levels by c-Myc KD; the levels of cleaved PARP remained unchanged (Fig 8B). c-Myc regulates the cell cycle through its effects on cdk inhibitors [21,22]. Consistent with these findings, c-Myc KD resulted in a slight, but consistent increase in p27 (Fig 8B). As expected, c-Myc KD resulted in reduced colony formation and inhibited cell growth in H441-FL cells under attached and detached conditions (Fig 8C and 8D).

We also examined the effects of c-Myc KD in H441, A549 and A549-FL cells. The results are shown in S5 Fig. c-Myc KD resulted in reduced colony formation and inhibited cell growth in H441 parental cells under attached and detached conditions. The inhibition was stronger under attached condition than under detached condition. c-Myc KD also resulted in reduced colony formation and inhibited cell growth in A549 and A549-FL cells; the inhibition was stronger under attached condition than under detached condition.

We then attempted to confirm the results of c-Myc KD by pharmacological inhibition using the prototype BET bromodomain inhibitor JQ1. JQ1 has been reported to exert prominent antitumor efficacy in a subset of non-small lung cancer cells harboring KRAS mutations through the down-regulation of Myc [16]. The results obtained are shown in Fig 8F and 8G. The treatment with 1 μM JQ1 lead to a slight decrease in c-Myc protein levels. In parallel, the stronger expression of p27 and enhanced apoptosis, as indicated by the induction of cleaved PARP, were observed following the treatment with 1 μM JQ1 (Fig 8E). We examined the effects of JQ1 on the growth of H441 and H441-FL cells under attached and detached conditions. Under attached conditions, JQ1 inhibited the growth of H441 and H441-FL cells to a similar extent (data not shown). Under detached conditions, JQ1 exerted similar inhibitory effects on the growth of both cell types; however, H441-FL cells were slightly more sensitive to 0.1 μM JQ1 than H441 cells (Fig 8F).

## Discussion

Decreased cell-substratum adhesion is assumed to play important roles in tumor metastasis. However, the mechanisms by which the loss of adhesion to the substratum leads to strong metastasis need to be validated and analyzed in an experimental model. Therefore, we herein examined the effects of a long-term exposure to the detached microenvironment in lung cancer cells. The lung cancer cells, A549 and H441, exposed to a long-term culture (up to 3–4 months) under 3D low-binding culture conditions gradually underwent morphological changes and acquired the ability to grow in suspension cultures. These FL cells exhibited greater metastatic potential in an intracardiac injection model of metastasis than parental cell lines. These FL cells differ from traditional non-adherent cells in their plasticity because FL cells readily attached and spread when plated on ordinary culture dishes. Thus, our results demonstrate that tumor cells, when exposed to long-term low cell-substratum adhesion, somehow adapt to grow in suspension cultures and, while doing so, acquire high metastatic capabilities *in vivo*.

The metastatic process consists of a sequence of several distinct steps: local cell growth, disengagement from the primary lesion with stromal destruction, vascular invasion, intravascular migration, adhesion to the vascular endothelium of the involved organ, invasion of the involved organ, and cell growth at the sites of metastasis [3]. In the present study, FL sublines showed greater cell growth ability in low attachment cultures than parental cell lines. This property may allow FL cells to survive and grow in stromal tissue fluid or within vascular and lymphatic spaces. Furthermore, FL cells may have an advantage in the early step of metastasis formation, during which cancer cells must survive and grow without firm anchorage to the matrix support in a new environment that is different from their primary site. These advantages may explain why FL sublines possess greater metastatic potential *in vivo* than parental cell lines.

We demonstrated using a whole genomic analysis, Western blot analysis, and knockdown experiments that the master regulator of EMT, ZEB1 may be causally involved in the high metastatic potential of A549-FL cells. A549-FL cells showed the stronger expression of ZEB1, and the knockdown of ZEB1 resulted in enhanced apoptosis, decreased cell colony formation, and decreased cell growth with or without attachment. EMT generally enables cells to gain a partial mesenchymal phenotype, in either a transient or permanent manner [23,24]. While many properties of cancer, including cell migration, invasion, metastasis, stem-like properties, and therapeutic resistance, have been ascribed to EMT [24,25], only a few previous studies have linked EMT or ZEB1 expression to cell growth in suspension cultures or anchorage-independent growth [26–28]. Takeyama et al. reported that ZEB1 KD suppressed the growth of ZEB1-high non-small lung cancer cells, particularly in a soft agar colony formation assay [29]. Using a Trk-induced EMT model in rat epithelial cells, Smit and Peeper also demonstrated that the depletion of ZEB1 caused apoptosis in suspension cultures, while it had no effect on cell growth or survival under adherent conditions [30]. The results of the present study reinforce these findings and reaffirm the important role of ZEB1, i.e., the promotion of metastasis through enhanced cell growth and survival under detached conditions.

In another KRAS mutant, H441, we did not find any evidence of EMT or ZEB1 overexpression as a feature of FL cells. A genome-wide copy number analysis indicated that the amplification of c-Myc was causally involved in the highly metastatic potential of H441-FL. c-Myc is a proto-oncogene that is located on chromosome 8q24 and codes for a transcription factor. The c-Myc protein regulates the general characteristics of tumorigenesis such as proliferation, survival, differentiation, and genetic stability, promotes cell cycle progression, and suppresses the cdk inhibitors, p21 and p27 [21,22]. Consistent with these findings, the knockdown of c-Myc in H441-FL cells led to the down-regulation of p27 and inhibition of cell growth in attachment and suspension cultures in our analysis. We also confirmed our results using the pharmacological inhibition of c-Myc with the bromodomain inhibitor JQ1, which exerted similar inhibitory effects to genetic knockdown on the growth of H441-FL cells. In contrast to the knockdown of c-Myc, JQ1 additionally induced apoptosis in H441-FL cells. This may be explained by the previous finding that JQ1 inhibits targets other than c-Myc [31].

Anoikis is programmed cell death induced upon cell detachment from the extracellular matrix [32]. We initially hypothesized that FL sublines developed resistance to anoikis in long-term low attachment cultures. Unexpectedly, the basal levels of apoptosis were higher in FL cells than in parental cells under detached conditions. These results suggest that the high growth rate of FL cells in suspension cultures is mediated by a high cell proliferation rate that exceeds elevated apoptotic levels. Among the various cell cycle regulators identified to date, the down-regulation of p27 was noted as a prominent feature of FL cells under detached conditions. Since p27 is a direct target of c-Myc, the c-Myc-p27 pathway may provide a mechanistic link for how H441-FL cells grow well in suspension cultures and exhibit high metastatic capabilities. The growth rate of A549-FL cells was significantly higher than that of H441-FL cells (Fig 2B and 2D). This result appears to contrast with the high p27 level of A549-FL cells vs. H441-FL cells. However, since the cell growth rate is governed by multiple cell cycle regulators, such as cyclins, cdks, and cdk inhibitors [33], it is not directly proportional to p27 levels in different cell lines. A previous pathological study reported a positive correlation between the expression of c-Myc and micropapillary lung adenocarcinoma, a histological subtype characterized by FL cancer cell clusters in tissues [9]. Thus, future studies are needed in order to investigate the role of c-Myc in cell growth under detached and metastatic conditions.

Molecules that confer growth advantages under detached conditions may also play important roles in cell growth under attached conditions; in the present study, the lentiviral knockdown of ZEB1 and c-Myc inhibited the growth of A549-FL and H441-FL cells, respectively, under attached and detached conditions. Furthermore, colony formation and cell growth were inhibited by ZEB1 KD in A549 cells and by c-Myc KD in H441, A549, and A549-FL cells. These results imply that drivers that play essential roles in cell growth under attached conditions may, when further overexpressed, confer cells with growth advantages under detached conditions. In support of this, we encountered difficulties obtaining A549-FL cells with stable ZEB1 KD and H441-FL cells with stable c-Myc KD, which may reflect the strong inhibitory effects of ZEB1 and c-Myc KD in cell growth and colony formation assays (Figs 7C, 7D, 8C and 8D). This hampered our investigation on the effects of stable ZEB1 KD and c-Myc KD *in vivo*.

It may be counterintuitive that no significant differences were observed in migration or invasion between A549-FL cells and parental A549 cells. Moreover, the invasion capacity of H441-FL cells was weaker than that of parental H441 cells. However, since the metastasis cascade involves multiple steps including invasion, cell growth, survival, immune evasion, and angiogenesis [3], it is conceivable that not all these parameters change in parallel in metastatic cells. For example, although the oncogenic roles of c-Myc are well established, c-Myc may exert differential effects on cell proliferation and migration in a cell context-dependent manner [34].

In conclusion, we herein presented data suggesting that long-term 3D low attachment cultures may hold promise as a useful and efficient method for obtaining cells with elevated cell growth in detachment cultures and high metastatic potential. We also believe that this method may have potential as a convenient method for elucidating the molecular mechanisms underlying metastasis mediated by decreased cell-substratum adhesion.

## Supporting information

S1 Fig Morphological comparison of parental and FL sublines before and after transduction of the firefly luciferase vector. (A) A549 and A549-FL cells, and (B) H441 and H441-FL cells did not reveal any significant changes after transduction of the firefly luciferase vector.
(TIF)

S2 Fig Observation period of murine metastasis models (n = 5) after an intracardiac tumor injection. In mice injected with luc-A549-FL cells, due to increased tumor burden and the loss of body weight, mice had to be sacrificed 1 week earlier on average than those injected with luc-A549 parental cell lines; however, this difference was not significant (A549, mean 41.4 days; A549-FL mean 34.2 days). Observation periods were similar in mice injected with luc-H441 and luc-H441-FL cells (H441, mean 54.6 days; H441-FL mean 55.2 days). The Data shows the box-and-whisker plot of the observation period after the injection (n = 5). Statistical analysis was performed by the Mann-Whitney U test.
(TIF)

S3 Fig Scatter diagram of signal levels by a gene expression array (A549-FL vs A549, H441-FL vs H441). The signal ratio of FL sublines to parental cell lines becomes larger toward the upper left of the diagram.
(TIF)

S4 Fig ZEB1 knockdown (KD) in A549 cells. (A, B) A549 cells were transduced with lentiviral vectors (shZEB1-A, shZEB1-B, and shScramble), and after a brief selection with puromycin, used for colony formation assay and cell count assay, without cloning. (A) Colony formation was inhibited by shZEB1-B, but not by shZEB1-A. (B) Cell growth was inhibited by shZEB1-B both in attachment and detachment cultures, but not by shZEB1-A. The data are shown as the box-and-whisker plot of 10 replicates. Statistical analysis was performed by the Kruskal-Wallis test followed by Tukey's test.
(TIF)

S5 Fig C-Myc knockdown (KD) in H441, A549 and A549-FL cells. (A) c-Myc expression was slightly increased in A549-FL sublines at the protein level. (B, C) H441 cells were transduced with lentiviral vectors (shc-Myc-A, shc-Myc-B, and shScramble), and after a brief selection with puromycin, used for colony formation assay and cell count assay, without cloning. (B) Representative images of macroscopic colony formation assay of 4 replicates of cultures (Giemsa stain). Colony formation was inhibited by shc-Myc-A and shc-Myc-B. (C) Cell growth was inhibited by shc-Myc-A and shc-Myc-B both in attachment and detachment cultures. Data are shown as the box-and-whisker plot of 10 replicates. (D, E) A549 and A549-FL cells were transduced with lentiviral vectors (shc-Myc-A, shc-Myc-B, and shScramble), and after a brief selection with puromycin, used for colony formation assay and cell count assay, without cloning. (D) Representative images of macroscopic colony formation assay of 4 replicates of cultures (Giemsa stain). Colony formation was inhibited by shc-Myc-A and shc-Myc-B in both A549 and A549-FL. (E) Cell growth was inhibited by shc-Myc-A and shc-Myc-B in attachment and detachment cultures in both A549 and A549-FL. The inhibition was stronger in parental A549 cells than in A549-FL cells. Data are shown as the box-and-whisker plot of 10 replicates. Statistical analysis was performed by the Kruskal-Wallis test followed by Tukey's test.
(TIF)

S1 Table A list of gene sets and gene symbols elected by GSEA (A549-FL vs A549). (XLSX)

S2 Table A list of gene sets and gene symbols elected by GSEA (H441-FL vs H441). (XLSX)
