# Supplementary figures and images for: Establishment of highly metastatic KRAS mutant lung cancer cell sublines in long-term three-dimensional low attachment cultures
Source: PLoS One. 2017 Aug 7;12(8):e0181342. doi: 10.1371/journal.pone.0181342 (PMC5546599; doi:10.1371/journal.pone.0181342)

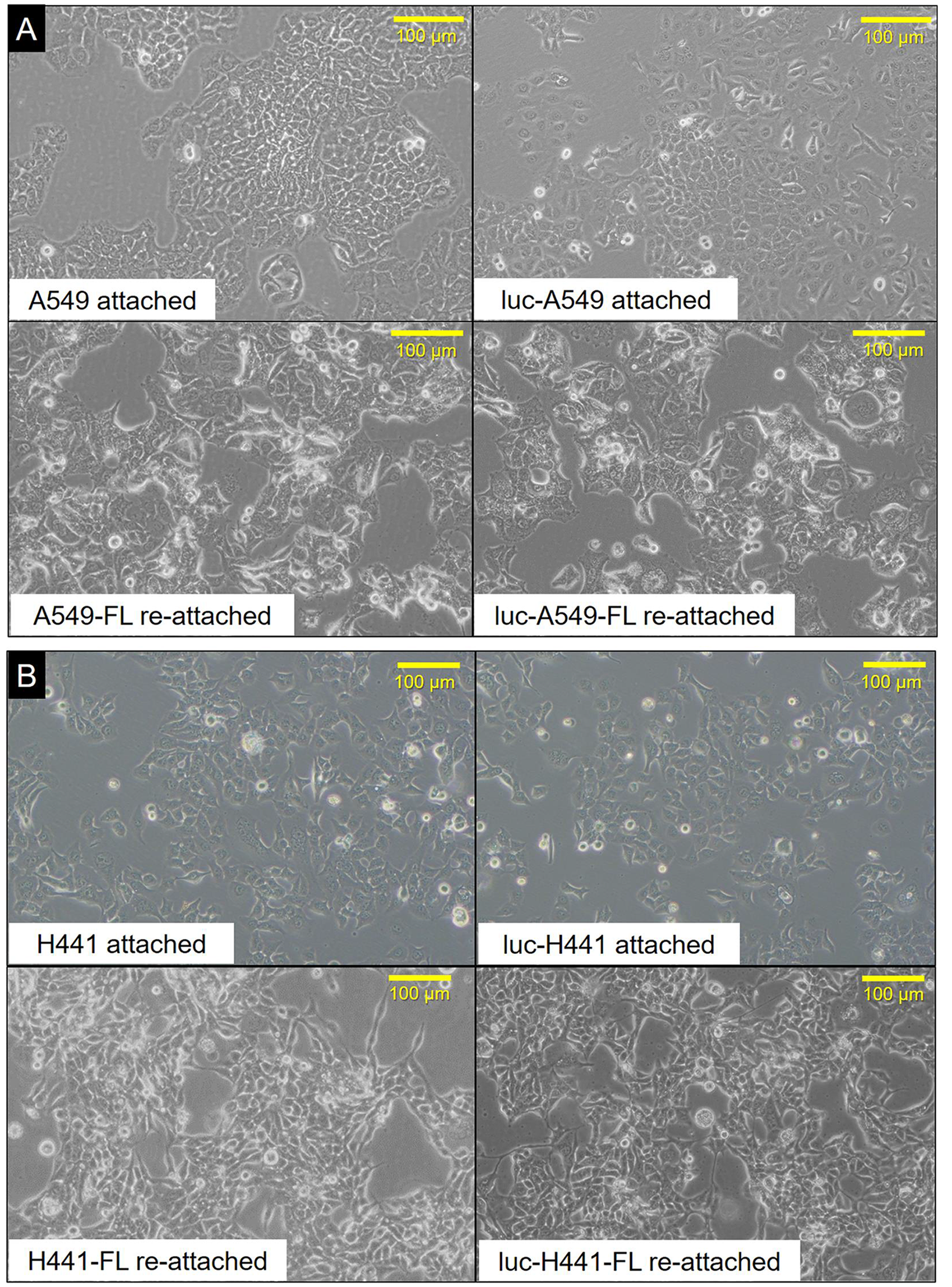

Supplement: S1 Fig — (A) A549 and A549-FL cells, and (B) H441 and H441-FL cells did not reveal any significant changes after transduction of the firefly luciferase vector. (TIF) [file pone.0181342.s001.tif]

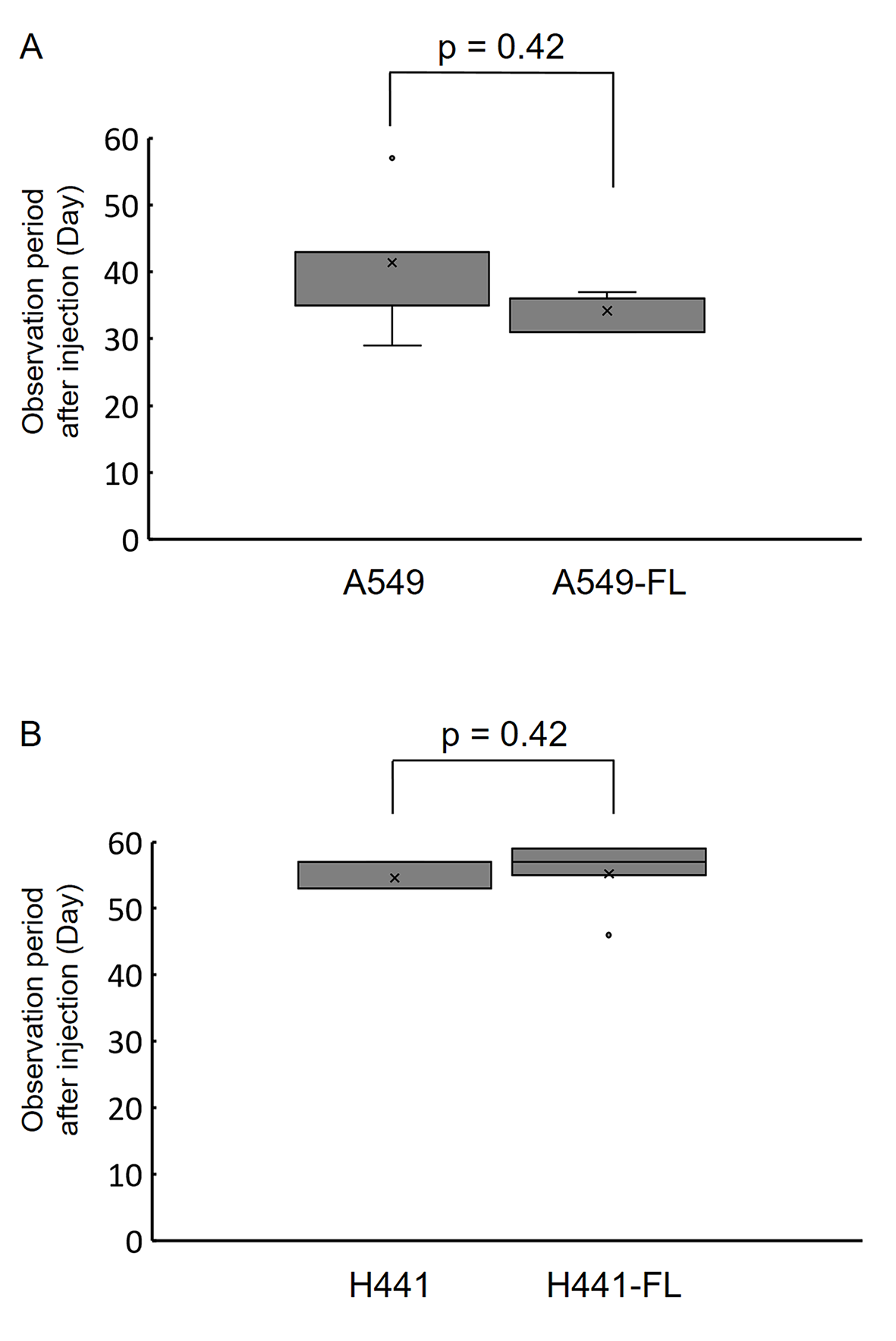

Supplement: S2 Fig — In mice injected with luc-A549-FL cells, due to increased tumor burden and the loss of body weight, mice had to be sacrificed 1 week earlier on average than those injected with luc-A549 parental cell lines; however, this difference was not significant (A549, mean 41.4 days; A549-FL mean 34.2 days). Observation periods were similar in mice injected with luc-H441 and luc-H441-FL cells (H441, mean 54.6 days; H441-FL mean 55.2 days). The Data shows the box-and-whisker plot of the observation period after the injection (n = 5). Statistical analysis was performed by the Mann-Whitney U test. (TIF) [file pone.0181342.s002.tif]

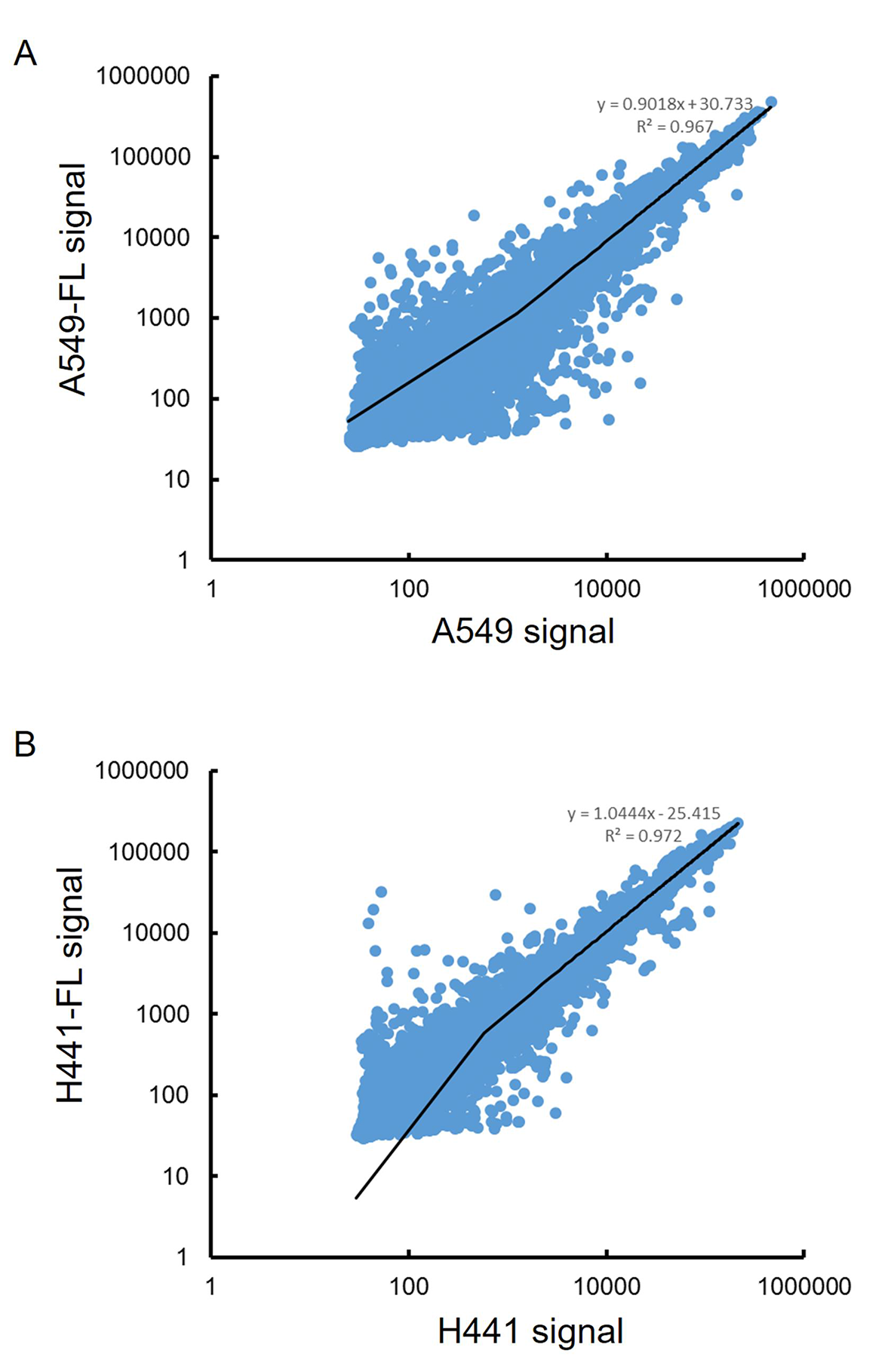

Supplement: S3 Fig — The signal ratio of FL sublines to parental cell lines becomes larger toward the upper left of the diagram. (TIF) [file pone.0181342.s003.tif]

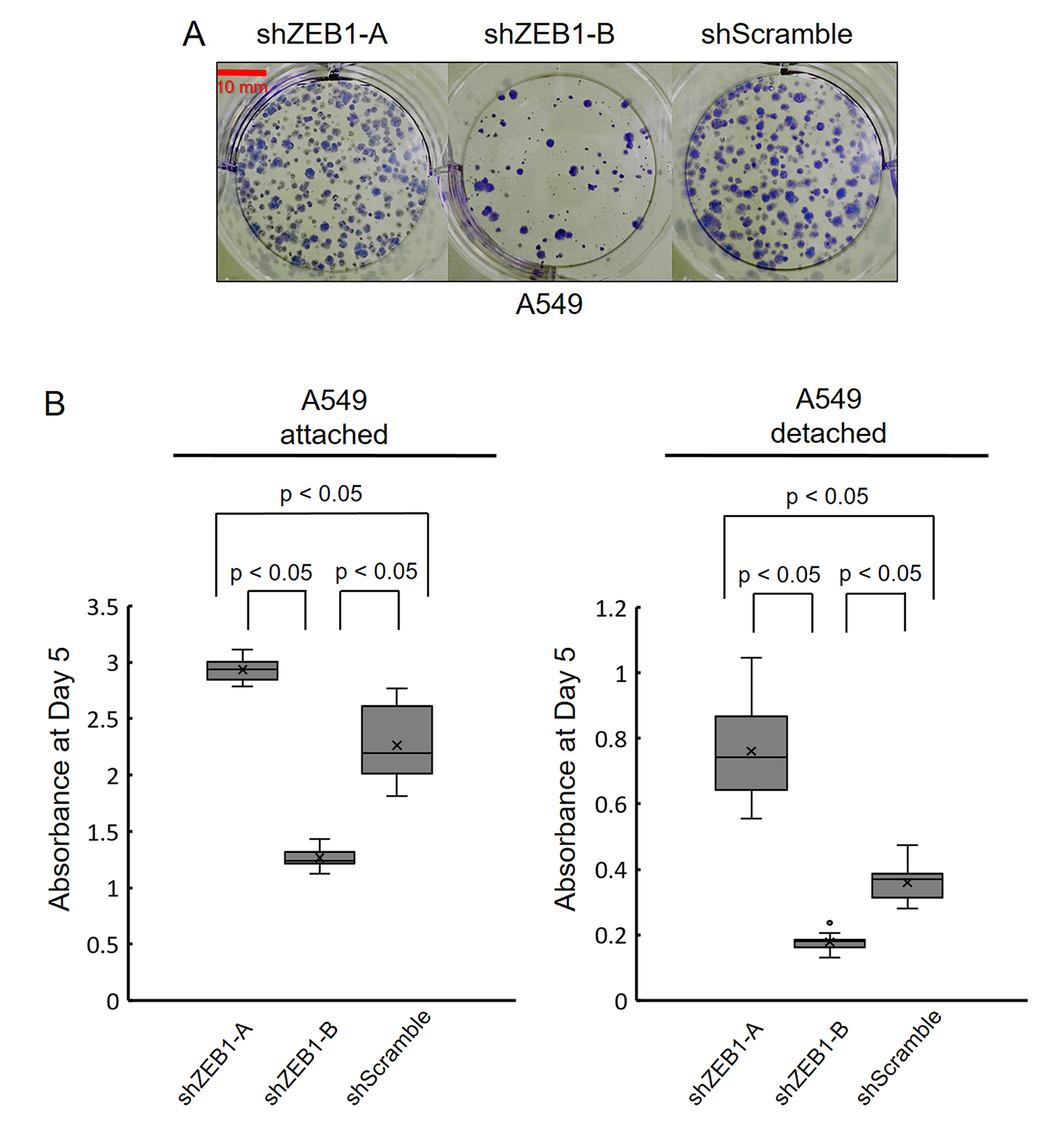

Supplement: S4 Fig — (A, B) A549 cells were transduced with lentiviral vectors (shZEB1-A, shZEB1-B, and shScramble), and after a brief selection with puromycin, used for colony formation assay and cell count assay, without cloning. (A) Colony formation was inhibited by shZEB1-B, but not by shZEB1-A. (B) Cell growth was inhibited by shZEB1-B both in attachment and detachment cultures, but not by shZEB1-A. The data are shown as the box-and-whisker plot of 10 replicates. Statistical analysis was performed by the Kruskal-Wallis test followed by Tukey's test. (TIF) [file pone.0181342.s004.tif]

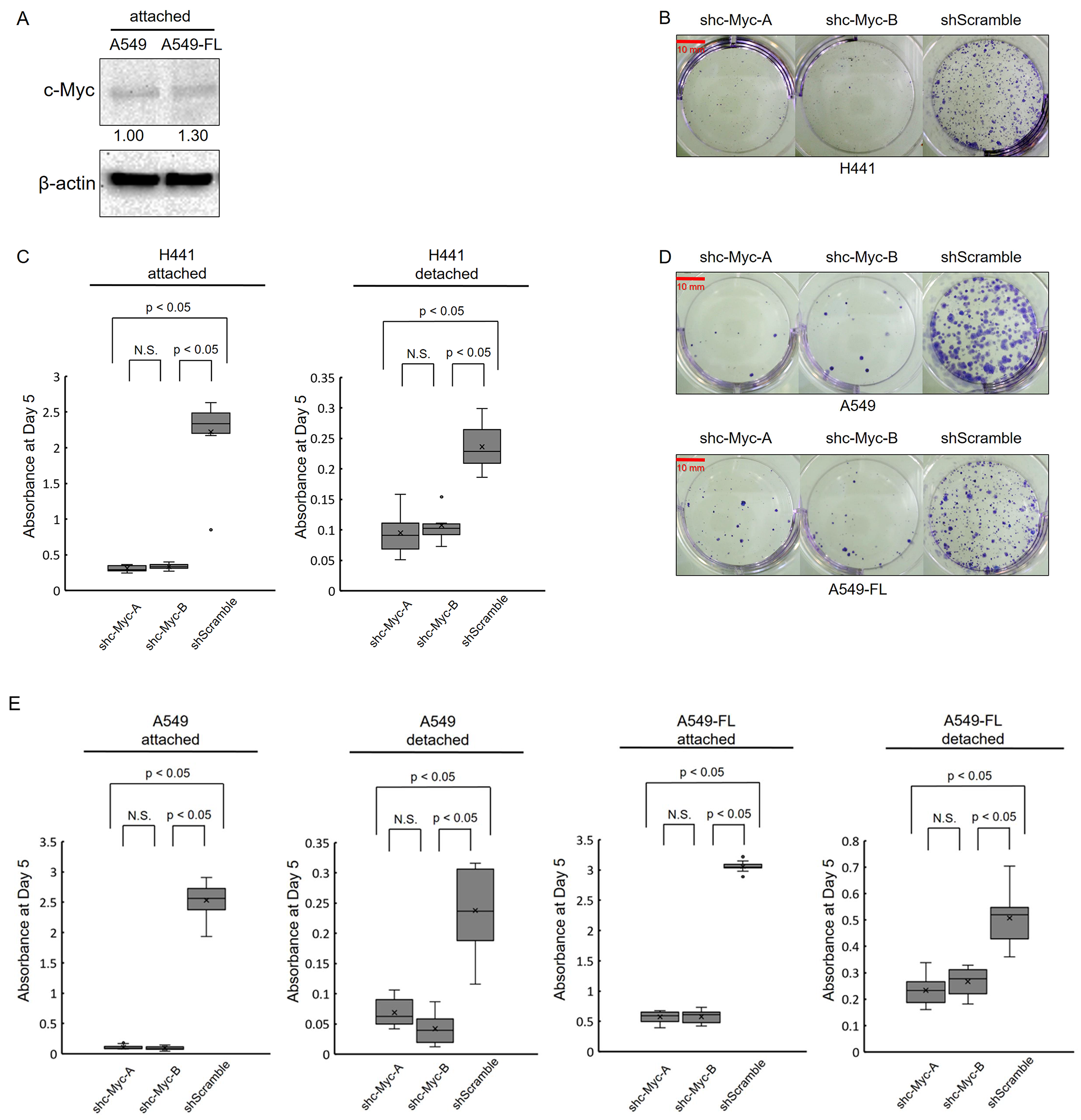

Supplement: S5 Fig — (A) c-Myc expression was slightly increased in A549-FL sublines at the protein level. (B, C) H441 cells were transduced with lentiviral vectors (shc-Myc-A, shc-Myc-B, and shScramble), and after a brief selection with puromycin, used for colony formation assay and cell count assay, without cloning. (B) Representative images of macroscopic colony formation assay of 4 replicates of cultures (Giemsa stain). Colony formation was inhibited by shc-Myc-A and shc-Myc-B. (C) Cell growth was inhibited by shc-Myc-A and shc-Myc-B both in attachment and detachment cultures. Data are shown as the box-and-whisker plot of 10 replicates. (D, E) A549 and A549-FL cells were transduced with lentiviral vectors (shc-Myc-A, shc-Myc-B, and shScramble), and after a brief selection with puromycin, used for colony formation assay and cell count assay, without cloning. (D) Representative images of macroscopic colony formation assay of 4 replicates of cultures (Giemsa stain). Colony formation was inhibited by shc-Myc-A and shc-Myc-B in both A549 and A549-FL. (E) Cell growth was inhibited by shc-Myc-A and shc-Myc-B in attachment and detachment cultures in both A549 and A549-FL. The inhibition was stronger in parental A549 cells than in A549-FL cells. Data are shown as the box-and-whisker plot of 10 replicates. Statistical analysis was performed by the Kruskal-Wallis test followed by Tukey's test. (TIF) [file pone.0181342.s005.tif]
